# Molecular basis for transfer RNA recognition by the double-stranded RNA-binding domain of human dihydrouridine synthase 2

**DOI:** 10.1093/nar/gky1302

**Published:** 2019-01-03

**Authors:** Charles Bou-Nader, Pierre Barraud, Ludovic Pecqueur, Javier Pérez, Christophe Velours, William Shepard, Marc Fontecave, Carine Tisné, Djemel Hamdane

**Affiliations:** 1 Laboratoire de Chimie des Processus Biologiques, CNRS-UMR 8229, Collège De France, Université Pierre et Marie Curie, 11 place Marcelin Berthelot, 75231 Paris Cedex 05, France; 2 Institut de biologie physico-chimique (IBPC), CNRS, UMR 8261 CNRS/Université Paris Diderot, 13 rue Pierre et Marie Curie, Paris 75005, France; 3 Laboratoire de cristallographie et RMN biologiques, UMR 8015, CNRS, Université Paris Descartes, Sorbonne Paris Cité, Paris, France; 4 Synchrotron Soleil, L’Orme des Merisiers, BP 48, 91192 Gif sur Yvette Cedex, France; 5 Macromolecular interaction platform of I2BC, UMR9198, Centre de Recherche de Gif-sur-Yvette, France

## Abstract

Double stranded RNA-binding domain (dsRBD) is a ubiquitous domain specialized in the recognition of double-stranded RNAs (dsRNAs). Present in many proteins and enzymes involved in various functional roles of RNA metabolism, including RNA splicing, editing, and transport, dsRBD generally binds to RNAs that lack complex structures. However, this belief has recently been challenged by the discovery of a dsRBD serving as a major tRNA binding module for human dihydrouridine synthase 2 (hDus2), a flavoenzyme that catalyzes synthesis of dihydrouridine within the complex elbow structure of tRNA. We here unveil the molecular mechanism by which hDus2 dsRBD recognizes a tRNA ligand. By solving the crystal structure of this dsRBD in complex with a dsRNA together with extensive characterizations of its interaction with tRNA using mutagenesis, NMR and SAXS, we establish that while hDus2 dsRBD retains a conventional dsRNA recognition capability, the presence of an N-terminal extension appended to the canonical domain provides additional residues for binding tRNA in a structure-specific mode of action. Our results support that this extension represents a feature by which the dsRBD specializes in tRNA biology and more broadly highlight the importance of structural appendages to canonical domains in promoting the emergence of functional diversity.

## INTRODUCTION

Splicing, nucleotides editing and post-transcriptional chemical modifications are important for the activation of RNA molecules (1–3). All these cellular reactions are catalyzed by specific enzymes. In that respect, how these enzymes specifically recognize and discriminate their substrate from the large cellular pool of ribonucleic acids still remain challenging questions in RNA-biology. One solution that nature has developed to satisfy this vital evolutionary pressure is the elaboration of several RNA-binding domains specialized in the recognition of various single or double-stranded RNAs (dsRNA) (4,5). Among these domains, dsRNA binding domain (dsRBD), already present in the last universal common ancestor (6), is extensively used by RNA metabolism enzymes likely due to its ability to function on its own and/or cooperate with multiple domains (7,8). This domain, typically ∼68 amino acids, is well-known for its functional versatility by means of a particular α1-β1β2β3-α2 canonical structure that allows it to recognize a variety of simple RNA structures ranging from A-form RNA helices to hairpins or tetraloops in shape-dependent manners (7,9,10), even though a sequence-specific mode of recognition has been invoked for a few of them (11,12). Remarkably, some proteins carry multiple copies of dsRBD in tandem to allow dynamic recognition of the RNA target since this domain is able to diffuse along dsRNA via an ATP independent process (13,14). In addition, the role played by dsRBDs is not limited to RNA recognition since it can serve in some cases as a platform for protein/protein interactions, including self-association (15,16). Beyond the canonical structure, there are several dsRBDs that host structural extensions attached to its N- and/or C-terminus (10,15). Unfortunately, at this stage, most of them remain unexplored. However, in the reported cases, these extensions have favored emergence of new functions (17–20). Several old and more recent NMR and crystallographic structures of dsRBDs from various proteins in complex with dsRNA made it possible to clarify how these domains function (11,12,21–27). In a generally quite conserved mode of action, helix α1 and loop β1–β2 recognize ribose moieties located within the dsRNA’s minor grooves (mG) while the N-terminal tip of helix α2 interacts with the phosphate backbone aligned along the dsRNA’s major groove (MG). Such type of recognition meets the requirements of many enzymes and proteins acting on long RNAs generally lacking a complex structure such as messenger and regulatory RNAs.

**Figure 1.**
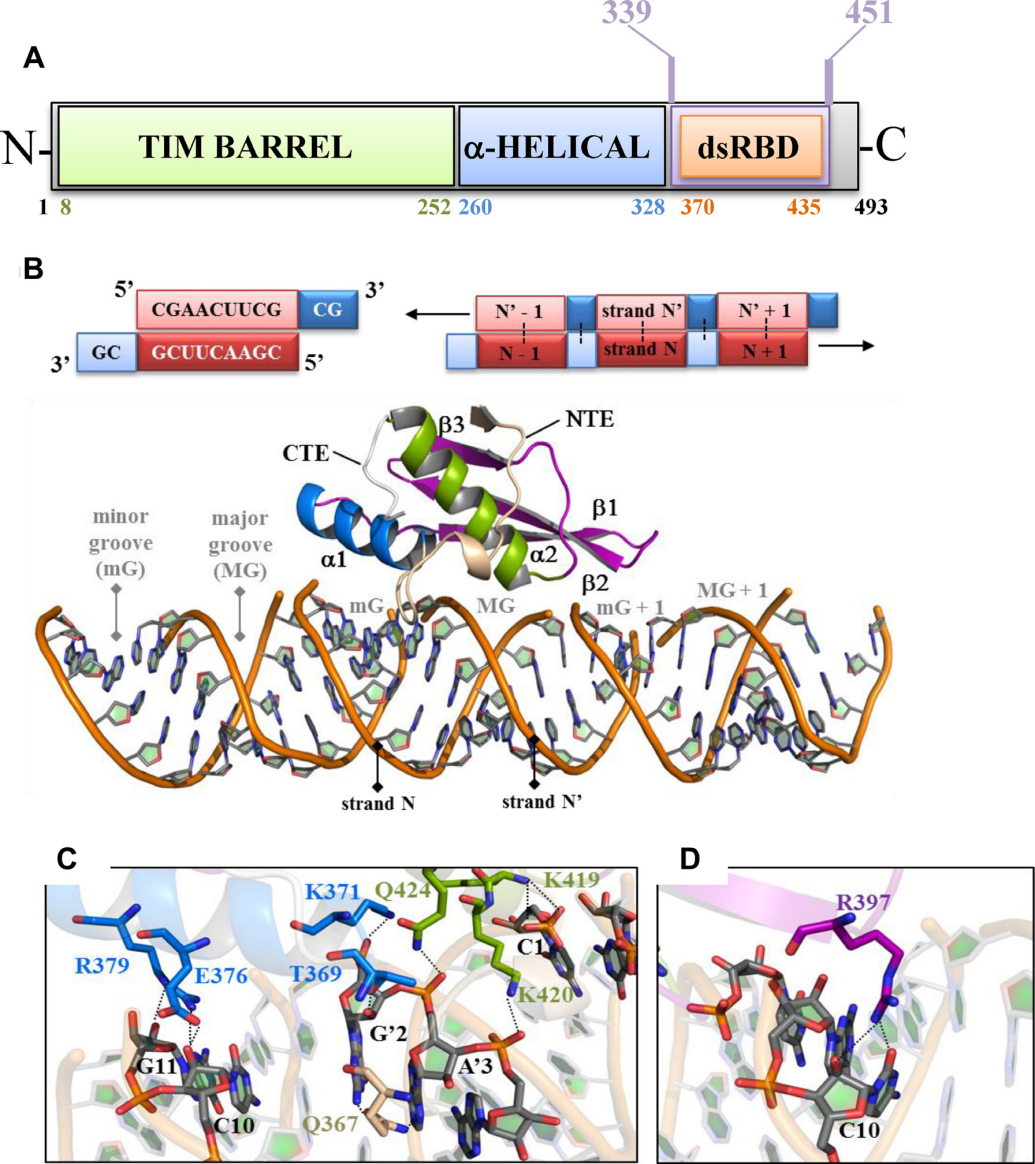
Mapping dsRBD residues involved in dsRNA recognition by X-ray crystallography. (**A**) Domain modularity of hDus2. In green is the TIM Barrel domain (TBD), which carries FMN, α-helical domain (HD) is in blue and the extended dsRBD is in purple. Within the latter, there is the canonical dsRBD in orange. (**B**) On top is shown the RNA palindromic sequence (5′CGAACUUCGCG3′) containing two overhang nucleotides used to generate a dsRNA by self-hybridization. The schematic representation of RNA palindromic sequence and two overhang nucleosides are shown as red and blue boxes, respectively. The 3′–5′ RNA and its 5′–3′ complementary strand are denoted as N and N′ respectively. Dots show the hybridization interface between two complementary sequences while the arrows indicate the 1D direction of macromolecular self-assembly of two complementary strands into a dsRNA helix-A structure. On the bottom is shown the X-ray structure of hDus2 dsRBD in complex with dsRNA. The protein is represented in cartoons wherein helix α1, α2 and β sheet of dsRBD are colored in blue, green and purple, respectively. The N-terminal (NTE) and C-terminal extensions (CTE) are colored in wheat and grey, respectively. dsRNA phosphodiester backbone is represented as an orange ribbon while the ribose and nucleobases are shown as sticks (gray). (**C**) View on the interactions between (i) residues of helix α1 (blue sticks) and NTE (wheat sticks) and nucleoside from the minor groove (mG) and helix α2 (green sticks) with nucleoside from the major groove (MG) of the dsRNA. The nitrogen, oxygen and phosphate atoms in protein or RNA are in blue, red and orange, respectively. (**D**) View on the interaction involving R397 (purple sticks) in the C-terminal end of the β1–β2 loop and nucleosides in MG + 1.

We and others have recently challenged this consensus belief with the discovery of an animal tRNA-modifying enzyme that carries a dsRBD, namely the dihydrouridine synthase 2 (Dus2) (28,29). Dus2 is found in all eukaryotes and catalyzes formation of dihydrouridine 20 in tRNA (Supplementary Figure S1A) (29–31). In addition to this physiological function, human dihydrouridine synthase Dus2 (hDus2) has been shown to promote certain cancers through its ability to interact with other enzymes, notably the aminoacyl tRNA synthetase complex EPRS and the protein kinase R, according to a mechanism that remains to be established (28,32). Like most Dus enzymes, plant and fungi Dus2 as well as the bacterial orthologue DusA carry an N-terminal TIM Barrel catalytic domain (TBD) in which the redox coenzyme FMN lies in the center of the barrel and a C-terminal helical domain (HD), the latter being considered as the main tRNA binding domain of Dus enzymes (30,31,33–36). However, in stark contrast, we showed that, despite the presence of a HD domain, hDus2 and by inference from the sequences probably all animal ones instead use a novel extended dsRBD version flanked by a peculiar N-terminal extension (NTE) and C-terminal extension (CTE) (Figure 1A, Supplementary Figures S1B and S2) to provide the major binding sites for tRNA substrate (29,36). In addition, hDus2 ends with ∼40–50 amino-acids predicted as intrinsically disordered and not involved in tRNA recognition. Hence, the NTE-dsRBD-CTE combination acts as a new prototype of tRNA binding module (Figure 1A and Supplementary Figure S1B). This finding raised the possibility that dsRBD could have eventually evolved to recognize an RNA substrate displaying a complex tertiary structure (the famous L-shaped tridimensional structure).

In the present report, we investigate the RNA binding mechanism of hDus2 dsRBD. By getting a crystal structure of this dsRBD in complex with a dsRNA and characterizing the dsRBD/tRNA complex by NMR, SAXS and extensive mutagenesis, we provide evidence that this dsRBD is specialized to recognize a tRNA substrate thanks to its peculiar NTE. The latter provides specific residues which, in combination with those of the canonical structure, expand the RNA-binding interface enabling the newly evolved domain to bind tRNA. This nicely illustrates how nature opportunistically recycles canonical RNA-binding structures by implementing small structural elements that work as effectors to generate original recognition units with new substrate specificities.

## MATERIALS AND METHODS

### Protein purification

hDus2 or its dsRBD (T339-K451 construct) containing both the NTE and CTE was cloned in a pET11d vector between BamHI and NcoI and expressed in BL21(DE3)pLysS or BL21(DE3) respectively (Novagen) in LB medium and was purified as previously described (29). Point mutations were carried out with Q5^®^ Site-directed mutagenesis kit (New England BioLabs) following the recommended procedure and done on dsRDB-pET11d vector. The resulting mutants were purified with the same procedure as used for the wild type protein. For NMR experiments, cells containing the plasmid encoding the dsRDB were grown in M9 minimal medium supplemented with 1 g/l of ^15^NH_4_Cl for ^15^N-labeling or 1 g/l ^15^NH_4_Cl and 2 g/l ^13^C-glucose for ^13^C/^15^N-labeling. The labeled proteins were purified following the protocol used for unlabeled dsRDB

### RNA preparation

Homo sapiens tRNA^Lys3^ or its ^15^N-labeled form was expressed in *E. coli* (JM101TR strain) from a recombinant plasmid and purified as previously described (37). The palindromic RNA used for crystallization was transcribed in vitro using T7 polymerase and 0.6 μM of complementary DNA 5′-CGCGAAGTTCGTATAGTGAGTCGTATTA-3′ while the RNAs used for RNase protection assay were transcribed *in vitro* using T7 polymerase and 0.6 μM of complementary DNA 5′-TGGTGCGAATTCTGTGGATCGAACACAGGACCTCCAGATCTTCAGTCTGGCGCTCTCCCAACTGAGCTAAATCCGCTATAGTGAGTCGTATTA-3′ for tRNA and 5′-TGGTGCGAATTCTGTGGATCGAACACAGGACCTCCCTTCGGCGCTCTCCCAACTGAGCTAAATCCGCTATAGTGAGTCGTATTA-3′ for its truncated version tRNA^ΔACS^. Briefly, 0.6 μM of 5′-TAATACGACTCACTATA-3′ was added to allow polymerase binding. The reaction was carried for 4 h at 37°C in 40 mM Tris–HCl pH 8, 5 mM DTT, 1 mM spermidine, 0.01% triton; 10% PEG 8000, 30 mM MgCl_2_ and 4 mM XTP (X = A; U; G or C).The transcription was quenched by addition of 2.64 g of urea and the resulting mixture was separated on a 8 M urea 15% (19:1) PAGE. The extracted band was further purified via a HiTrapQ (qiagen) with a gradient of 300 mM sodium acetate to 3 M sodium acetate. Buffer was exchanged with a desalting PD-10 column (GE healthcare) and the RNA was annealed prior to use.

### Crystallization, data collection, structure determination and refinement

Crystals of dsRBD-dsRNA complex were obtained by mixing an equivalent ratio of protein:RNA in 50 mM Tris–HCl pH 8, 150 mM NaCl and 1 mM MgCl_2_ and left on ice for 1 h. The palindromic RNA sequence is 5′CGAACUUCGCG3′. This RNA carries two overhang nucleotides (CG) at its 3′ extremity, which is used to allow this single stranded RNA to self-assemble into a pseudo A-helix and promote crystal packing. 1 μl of freshly prepared complex was mixed with 1 μl of reservoir comprised of 15% isopropanol, 20 mM MgCl_2_ and 50 mM 2-(*N*-morpholino)ethanesulfonic acid (MES) pH 6 and were left to equilibrate by vapor diffusion method. Crystals grew after 1 month and were transferred in a solution identical to that of the reservoir with the addition of 30% PEG 400 prior to flash freezing with liquid nitrogen. R361A-R362A dsRBD mutant was crystallized by vapor diffusion against 22% PEG 8000 and 100 mM sodium cacodylate pH 6; K419A–K420A dsRBD mutant required 35% PEG 4000, 1 M lithium chloride and 100 mM Tris pH 8.5. Crystals were obtained after 3 days and were cryoprotected using 15% glycerol before flash freezing. Data collection, structure determination and refinement for the dsRBD-dsRNA complex and for the dsRBD double mutants were described in the supplementary materials.

### Binding assays

Electrophoretic mobility shift assays were carried for the dsRBD and its different mutants using a 6% native (19:1) PAGE at 4°C with 100 V. Increased concentrations of proteins were added to a fix concentration of Homo sapiens tRNA^Lys3^ (1 μM) and incubated at room temperature for 20 min in 50 mM Tris–HCl pH 8, 10% glycerol, 5 mM DTT and 150 mM ammonium acetate prior to migration. RNA was visualized by toluidine coloration and quantified using ImageJ (https://imagej.nih.gov/ij/index.html). The error bars are calculated from three independent sets of experiments. The *P* values for WT, R361A, R362A and R361A-R362A dsRBDs are 0.05, 0.0302, 0.0089 and 0.0366, respectively.

### RNase protection assays

20 μM of hDus2 was incubated on ice for 20 min with 10 μM of RNA in the same buffer as the binding assays, followed by the addition of 2.5 μl RNase A at 1 μg/ml. The reaction was quenched by adding 2% sodium dodecyl sulfate (SDS) and heated at 100°C for 2 min. Degradation pattern was analyzed on a denaturing 8% PAGE (19:1 acrylamide:Bis solution) with 8 M urea colored by toluidine.

### SEC-MALLS

Analysis were done by size-exclusion chromatography (SEC) on a Superdex 200 10/300 GL (GE Healthcare) using a Shimadzu Prominence HPLC. Multi-angle laser light scattering (MALLS) was measured with a MiniDAWN TREOS equipped with a quasi-elastic light scattering module and a refractometer Optilab T-rEX (Wyatt Technology). Protein concentration was determined using a specific refractive index (dn/dc) of 0.183 at 658 nm while for RNA the value was 0.17. Stoichiometry of the complexes was determined using the method protein conjugate.

### NMR experiments

All NMR spectra of dsRBD and tRNA were measured at 303 K on a Bruker AVIII HD 600 MHz equipped with a cryoprobe in a buffer containing 25 mM sodium phosphate pH 6.5, 20 mM sodium chloride, 10 mM dithiothreitol (DTT) and 10% (v/v) D_2_O. The data were processed using TOPSPIN 3.5 (Bruker) and analyzed with Sparky (www.cgl.ucsf.edu/home/sparky/). The backbone resonances of dsRBD (residues 337–450) were assigned using 2D (^1^H,^15^N)-HSQC, 3D HNCA, 3D HNCACB, 3D CBCA(CO)NH, 3D HNCO and 3D NOESY-(^1^H,^15^N)-HSQC. For titration experiments, 0.35 mM of ^15^N-labeled dsRBD were mixed with increasing amount of unlabeled tRNA^Lys3^ up to 1 molar equivalent and 2D (^1^H,^15^N)-HSQC were recorded at each step. Similarly, the titration of 0.3 mM ^15^N-labeled tRNA^Lys3^ was performed with 2D (^1^H,^15^N)-TROSY experiments by adding increasing amount of unlabeled dsRBD up to 1 molar equivalent.

### SAXS data collection, analysis and model generation

SAXS experiments were performed at the SWING beamline at the SOLEIL synchrotron (Saint-Aubin, France) using an online high-performance liquid chromatography (HPLC). SAXS data collection, analysis and model generation are described in the supplementary materials.

## RESULTS

### The dsRBD of hDus2 recognizes the dsRNA with an unprecedented mechanism

To investigate how this novel extended dsRBD recognizes a dsRNA molecule, we crystallized hDus2′s dsRBD (construct T339-K451 of human Dus2) in complex with an eleven palindromic oligo-ribonucleotide and solved the whole structure (Figure 1B and Supplementary Table S1). The designed dsRNA, which harbors two overhangs at the 3′-extremities, self-assembles into a pseudo A-helix (Figure 1B and Supplementary Figure S3). Overall, there are no major structural changes detected between free and bound dsRBD, except for a notable modification in the twist of the C-terminal region of strand β1 induced by dsRNA that consequently alters loop β1–β2 conformation (Supplementary Figure S4A and B). This conformational change does not appear to disrupt the conformation of aromatic residues Y388 in strand β1 and F399 in strand β2, known to be strictly conserved among dsRBDs and critical for stabilizing, with additional residues, the hydrophobic core of the canonical structure (Figure S4C).

Recognition of dsRNA is essentially achieved via three major canonical regions namely helix-α1, helix-α2 and the C-terminal part of the β1–β2 loop. Three residues of helix-α1 (T369, E376, R379) interact with ribose 2′-OH groups in dsRNA’s minor groove while K371 of helix-α1 together with K419, K420 and Q424 located in the N-terminal extremity of helix α2 recognize exclusively the phosphodiester backbone of the major groove (Figure 1c). Remarkably, the C-terminal part of the β1–β2 loop binds to the mG+1 minor groove via R397, which makes hydrogen bonds with both the ribose and nucleobase C10 (Figure 1D). To our knowledge, involvement of this region of the β1–β2 loop in direct contact with a dsRNA is quite unusual. Note that animal Dus2 dsRBDs have a shorter β1–β2 loop, with 2–3 residues less than the one of archetypal dsRBDs, preventing the canonical mode of RNA recognition via this loop, and likely explaining the peculiar contacts observed for this region in the dsRBD-RNA structure (see also the discussion).

Although the majority of dsRNA recognition is ensured by interactions involving the canonical fold, our structure reveals that there is a residue Q367 located in the NTE, more precisely between the 3_10_ helix and the beginning of helix-α1 that also participates to dsRNA recognition. Q367 interacts with C8, A’3 and G’2 in proximity to helix-α1 binding site further extending the interaction surface with the minor groove (Figure 1C). Collectively, the dsRBD of hDus2 has a dsRNA binding capacity *via* the cooperative action of both its canonical structure and NTE.

### The canonical region of the dsRBD and NTE jointly participate to tRNA recognition

**Figure 2.**
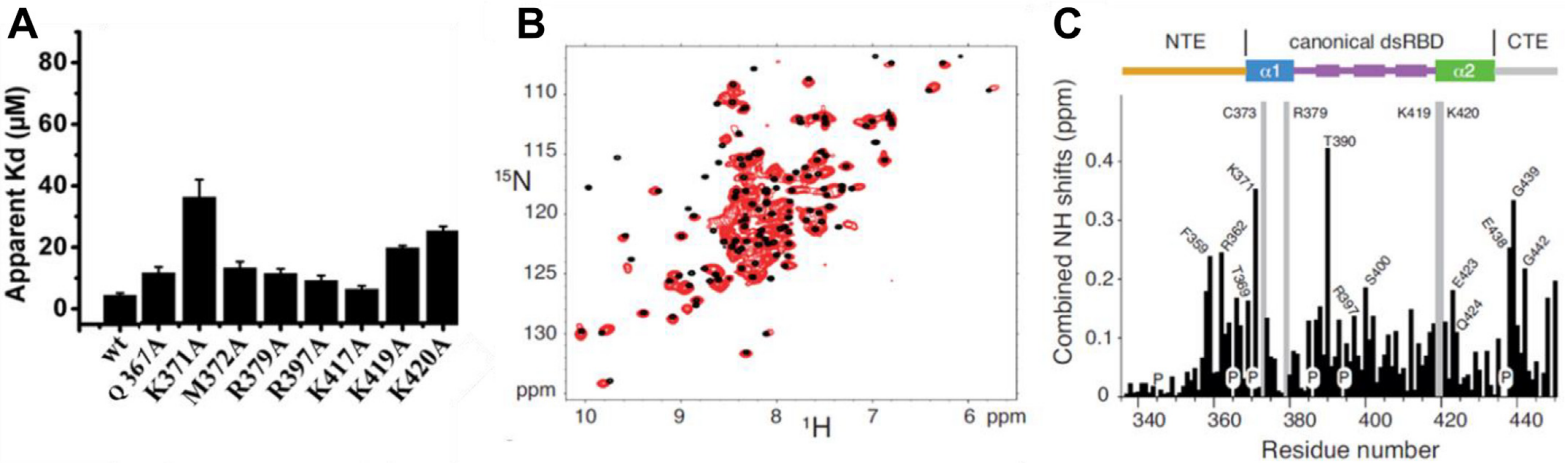
Characterization of dsRBD-tRNA complex by NMR and mutagenesis. (**A**) Histogram showing the apparent dissociation constant (*K*_d_) in μM of dsRBD and its mutants for human tRNA^Lys3^. (**B**) shows the (^1^H,^15^N)-HSQC experiments of ^15^N labeled dsRBD free in solution in black or with 1 equivalent of human tRNA^Lys3^ in red. (**C**) Chemical shift perturbation of dsRBD calculated as [(Δδ^15^N)^2^/7+(Δδ^1^H)^2^]^1/2^ between its free and tRNA-bound forms. Unobservable residues after titration are shown in grey. Missing values are from proline residues (P) or residues with missing assignment in the free form of dsRBD. The secondary structure of dsRBD is placed above the histogram.

dsRBD can bind an RNA harboring a canonical A-form helix, however, this domain should have a preference for a tRNA. To explore this issue, we first assessed if the residues implicated in dsRNA recognition are also involved in tRNA recognition by measuring apparent dissociation constant (Kd) of human tRNA^Lys3^ for several dsRBD alanine mutants via gel shift assay (Figure 2A and Supplementary Figure S5). Mutation of the conserved residue, K371, reduced the binding by ∼8-fold while that of K419 or K420 by >4-fold respectively, demonstrating the importance of interactions engaging both α1 and α2 helices. The C-terminal edge of the β1–β2 loop is also involved but to a lesser extent since R397A variant showed only a mild effect on affinity with a reduction of ∼2-fold. The results clearly showed that residues involved in dsRNA binding also participates to tRNA binding with an important contribution of K371, K419 and K420. Secondly, we compared the ability of hDus2′s dsRBD to discriminate tRNA from synthetic dsRNA and confirmed that it has a higher apparent affinity for tRNA (>3-fold) (Supplementary Figure S6A and B). This indicates that the interaction interface with tRNA is probably broader than that with a dsRNA. Finally, to delineate the functional tRNA binding site at the residue level, NMR spectroscopy titrations of unlabeled human tRNA^Lys3^ with ^15^N-labeled dsRBD was performed. Backbone resonance of wild type dsRBD was obtained using standard triple resonance experiments (Supplementary Figure S7A). The complex is in the intermediate exchange regime at the NMR chemical shift time scale yielding disappearance and re-appearance of the signals at a 1:1 ratio. Overall, peaks broadening as well as chemical shift displacement confirm formation of a dsRBD/tRNA complex (Figure 2B and C). Residues of helix α1 are involved in tRNA recognition since K371, T369 and L374 resonances are significantly shifted while R379 signal is lost and could not be identified in the bound form, confirming the involvement of the C-terminal edge of the β1–β2 loop in tRNA recognition. In addition, K417, S418, L421 and E423 are also affected while no signal could be assigned to K419 and K420 showing a strong implication of helix α2 in RNA binding. T390 amide group located in β1 exhibits a perturbation of 0.42 ppm most probably related to the modification in the twist of the C-terminal region of strand β1 upon RNA-binding as revealed in the crystal structures (Supplementary Figure S4). Additionally, some residues located at the C-terminus of the canonical domain, namely E438, G439, G442 and L448 are perturbed upon tRNA addition. These changes are most probably due to indirect effects rather than direct RNA contacts since this extremity interacts with helix α1 and is far from the RNA binding interface observed in our dsRBD/dsRNA crystal structure (Figure 1B). Remarkably, the signals of NTE residues, namely K358, F359, R362 and A366 are highly perturbed upon tRNA addition. This is again in agreement with the dsRBD/dsRNA crystal structure showing that NTE participates to minor groove recognition via Q367 (Figure 1C). Altogether, these results confirmed the implication of canonical dsRBD interactions in tRNA recognition but also that NTE is involved in direct contact with tRNA.

### NTE acts as a cooperative effector in dsRBD specialization toward tRNA recognition

**Figure 3.**
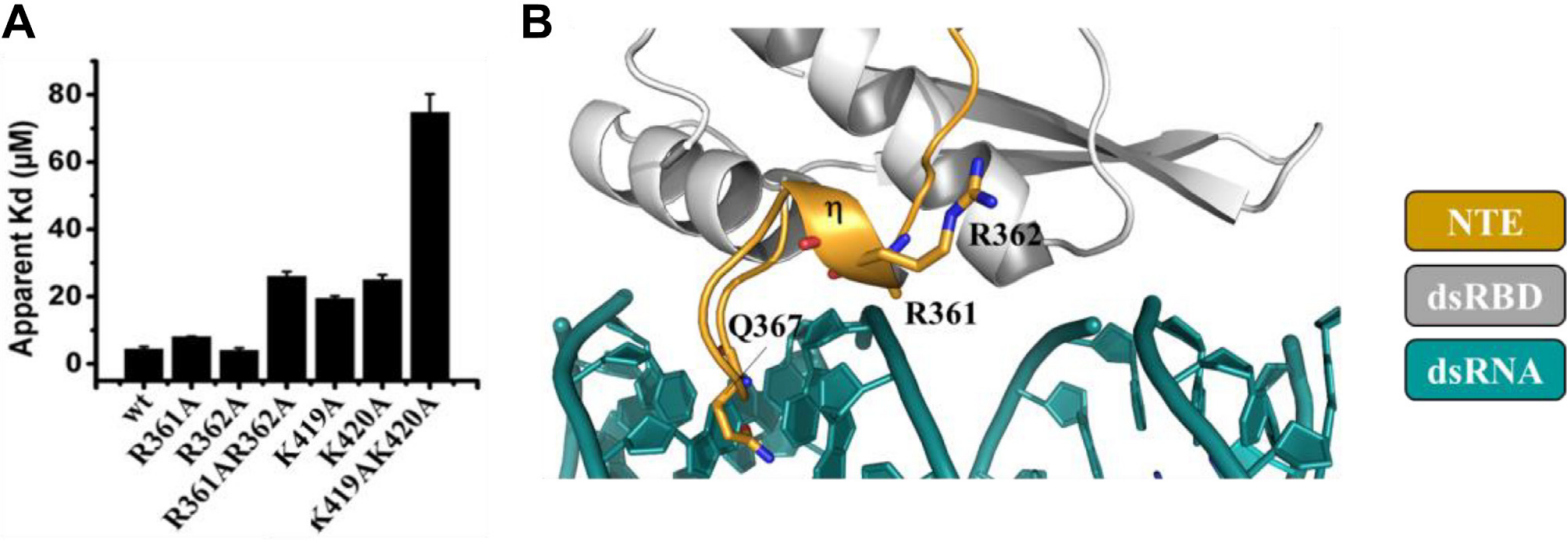
Synergetic action of NTE in tRNA recognition. (**A**) Histogram showing the apparent dissociation constant (Kd) in μM of dsRBD simple and double mutants for human tRNA^Lys3^. (**B**) View on the 3_10_ helix of NTE (orange) showing Q367 and the two arginine residues R361 and R362 as sticks. The density corresponding to R361 side chain is not observed indicating its flexible nature. The canonical dsRBD is in gray while the dsRNA is in dark green.

NMR identified R362, a conserved residue in NTE, as potentially involved in tRNA interaction, but curiously its mutation seems to have no impact (Figure 3A). A closer look at the sequence alignment revealed that the adjacent residue, R361, bears a positive charge and is also conserved (Supplementary Figure S2). Its mutation slightly reduced binding, however, the double mutant R361A-R362A exhibited a remarkable 6-fold lower binding capacity for tRNA (Figure 3A). To assess whether the reduced binding capacity of this double mutant is a direct effect of the missing positive charges and not a consequence of structural change, we solved its X-ray structure (Supplementary Table S1). As shown in Supplementary Figure S8A and B, no obvious changes are observed as evidenced by a RMSD of 0.18 Å over 82 Cα between the mutant and wild type structures. Thus, the results suggest that R361 and R362 act synergistically via electrostatic interaction with tRNA. Although, R361 and 362, both localized in NTE helix 3_10_, are not contacting the dsRNA, they are nonetheless relatively close suggesting that they recognize a structural element present in the tRNA and absent in dsRNA or they undergo a conformational change activated by the tRNA substrate itself. This is consistent with the fact that dsRBD’s affinity for tRNA^Lys3^ is ∼3-fold higher than that for dsRNA whereas R361A–R362A variant exhibits similar affinities for these two RNAs (Supplementary Figure S6I & J). In order to substantiate the idea of a synergistic effect due to NTE, we tested the effect of a double mutation in the canonical dsRBD. We chose to produce K419A–K420A because of the important role played by these residues (see above). As shown in Figure 3A, the double mutant led to a ∼17-fold decrease in the apparent affinity for tRNA, rather hitting for an additive mode of action of these residues. Similarly to R361A–R362A, this effect is purely attributed to loss of positive charges since the structure of K419A–K420A is similar to that of the wild type (RMSD of 0.6 Å over 82 Cα) (Supplementary Figure S8A and B). Finally, we also observed that Q367A mutant present an impaired ability to discriminate between a dsRNA and tRNA (Supplementary Figure S6G and H). Overall, our results indicate that NTE is involved in direct interaction with tRNA and contributes to discrimination of cognate RNA structure.

### Mapping dsRBD binding site on tRNA

**Figure 4.**
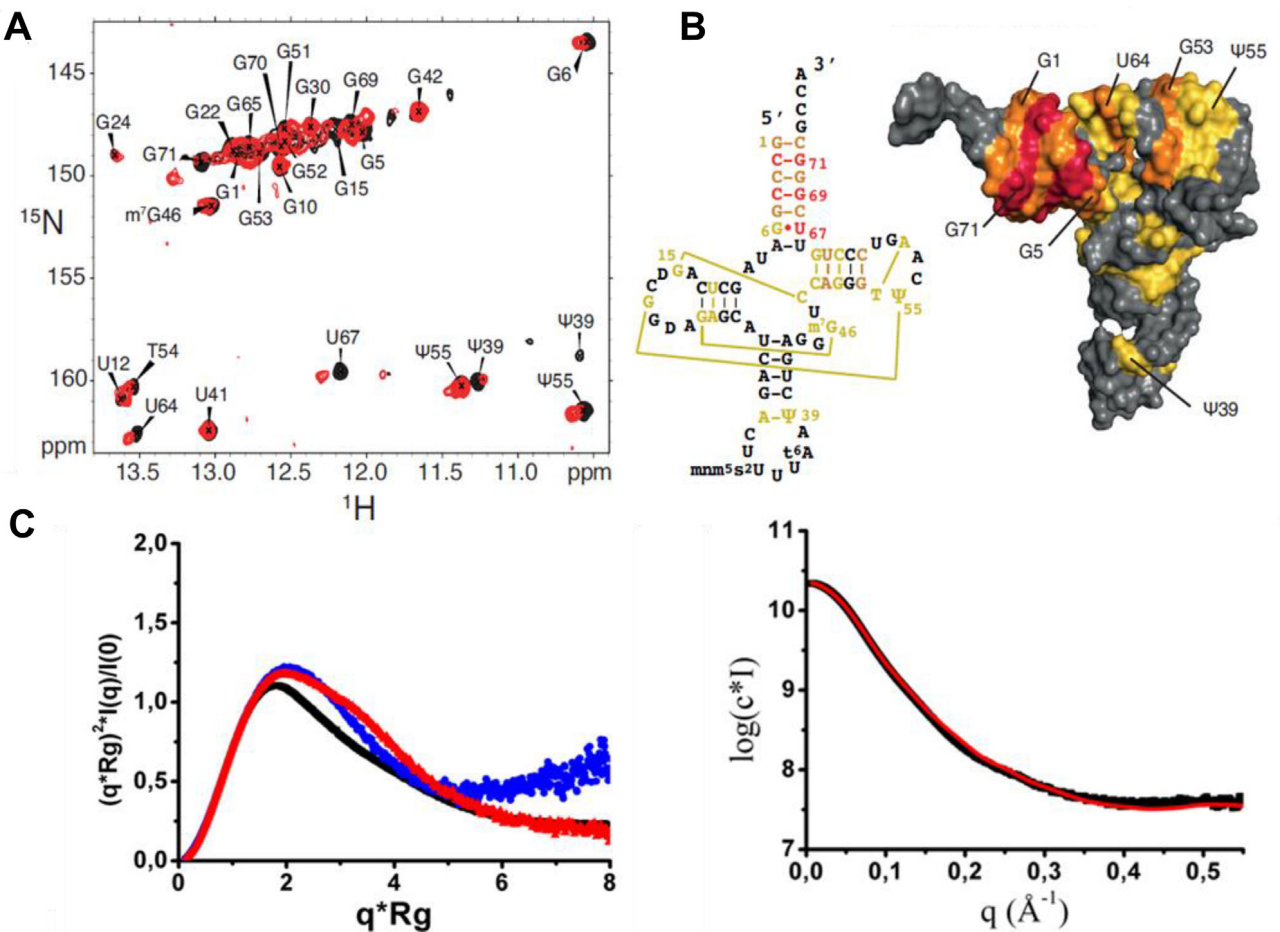
Identification of the binding interface of dsRBD on tRNA. (**A**) (^1^H,^15^N)-TROSY of ^15^N-labeled tRNA^Lys3^ in its free state (black) and in the presence of 1 equivalent of dsRBD (red). (**B**) *left:* secondary structure of human tRNA^Lys3^ showing the nucleotides that are affected upon dsRBD binding (strongly affected in red: Δδ > 0.15 ppm; moderately affected in orange: Δδ > 0.08 ppm; slightly affected in yellow: Δδ > 0.03 ppm). *right:* Representation on the surface of human tRNA^Lys3^ (pdb 1FIR) of the nucleotides affected upon dsRBD binding. The same color code is used. (**C**) SAXS characterization of dsRBD, human tRNA^Lys^_3_ and their reconstituted complex *in vitro*. Left: Normalized Kratky plot for tRBD (blue), tRNA^Lys^_3_ (red) and their complex (black). Right: ab initio multiphase reconstruction of tRBD-tRNA^Lys^_3_ complex SAXS data using a three-phase model by MONSA. The experimental curve is shown in black while in red is the theoretical curve from the MONSA model (χ^2^ = 3.78).

Next, NMR titration was performed with ^15^N-labeled tRNA^Lys3^ and unlabeled dsRBD (Figure 4A) to delineate the binding interface on tRNA. Resonances of tRNA^Lys3^ imino groups were assigned previously (38). Based on chemical shift perturbation, we were able to identify two distinct clusters of nucleosides impacted by dsRBD binding (Figure 4A and B). The first region involves the acceptor stem and the TΨC arm, their signals being significantly perturbed upon dsRBD addition, which indicates a strong binding. Namely, U67, G69 and G71 located in the acceptor stem are strongly impacted while G5 and G70 in that same region and G53 and U64 in the TΨC loop are also affected but to a lesser extent (Figure 4A and B). The second cluster includes U12 and G22 in the D stem as well as Ψ39 in the anticodon, but the amplitude of the perturbations associated with these nucleosides is weak, likely indicating an unspecific interaction site. To further investigate the dsRBD/tRNA^Lys3^ structure, SAXS measurements were performed on the individual components and on the complex (Figure 4C and Supplementary Figure S9A–C). Unbound dsRBD and tRNA^Lys3^ have an Rg of 18 and 23 Å, respectively (Supplementary Table S2). The reconstituted dsRBD/ tRNA^Lys3^ complex presents a higher Rg ∼29.6 Å while exhibiting a more globular shape than its individual components (Figure 4C, middle panel, and Supplementary Table S2). A*b initio* multiphase volumetric approach using MONSA was performed to resolve the individual components in the complex. This led to a model fitting the data with a χ^2^ of 3.78 suggesting small conformational differences between the bound and unbound forms (Figure 4C, right panel). In this model, one dsRBD binds near tRNA’s TΨC arm while the second one binds close to its anticodon arm bottom (Supplementary Figure S9G), which remarkably agrees with both NMR and SEC-MALLS data (Supplementary Figure S10). However, SEC-MALLS experiments done on full length hDus2 free and in complex with tRNA^Lys3^ revealed that the enzyme forms a monomer in solution and interacts with tRNA in a 1:1 molar ratio (Supplementary Figure S11). Thus, the presence of TBD + HD seems to increase hDus2 specificity probably by restricting the dsRBD positioning. Altogether, we propose that only the first site, located at the acceptor stem and T/Ψ stem-loop, is biologically relevant given its larger interface and its proximity to the target nucleotide modified by Dus2.

### dsRBD adopts a rigid conformation in hDus2

**Figure 5.**
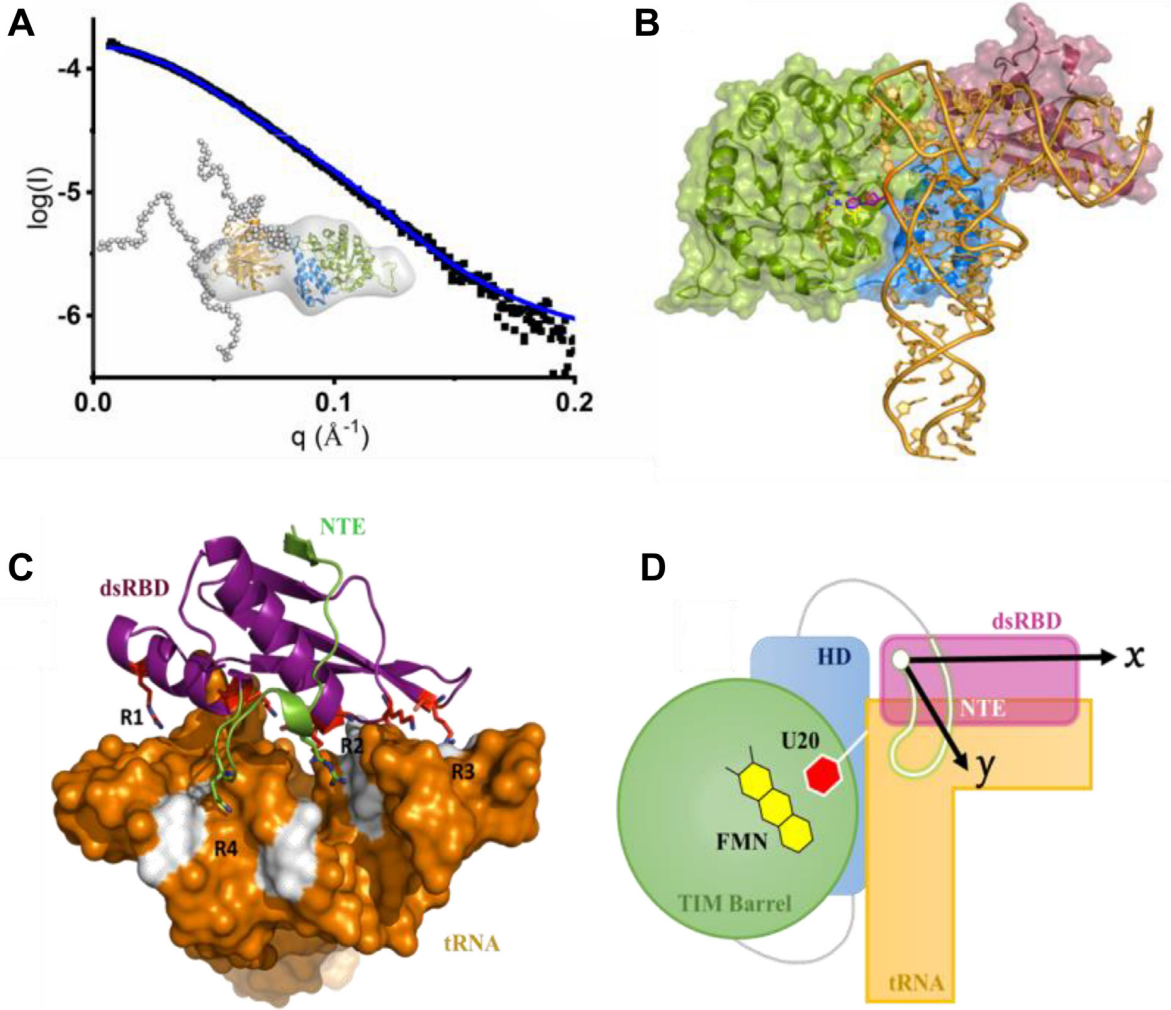
Molecular model of dsRBD/tRNA complex. (**A**) SAXS characterization of full length hDus2. Superposition of the experimental (black) and theoretical scattering curves (blue) of hDus2 model; insert: hDus2 model ensemble generated by eom from TBD+HD and dsRBD known X-ray structures superposed on the *ab initio* shape obtained from dammif. Color code is identical to Figure 1A. In dots are represented the constructed chains by eom. The minimal ensemble best fitting the curve is comprised of 3 components with both DusD and dsRBD having a fixed orientation while flexibility is observed for the C-terminal extremity. (**B**) Model of full length hDus2/tRNA complex. This model was generated by structural alignment of full length hDus2 with *Thermus thermophilus* orthologue DusA in complex with tRNA (PDB: 3B0V) (33). TIM Barrel domain, HD and dsRBD are in green, blue and violet respectively. (**C**) Postulated molecular model for dsRBD/tRNA that is inferred from (**C**) and supported by compelling biochemical and structural data gathered in this study. tRNA is shown as surface in orange while the canonical dsRBD and NTE are represented as violet and green cartoons, respectively. Colored in white are the nucleotides that are the most impacted by dsRBD binding determined from NMR chemical shift mapping. tRNA recognition by dsRBD is achieved *via* four regions: R1 to R3 involves the canonical dsRBD while R4 involves only the NTE. (**D**) Proposed schematic model of full length hDus2-tRNA complex showing the catalytic TIM Barrel domain wherein FMN prosthetic groups (yellow) and the flipped U20 target (red) lye in its center, as a green cycle, HD in blue, dsRBD in violet, NTE in green and tRNA in orange. The arrows show the 2D-surface screened by dsRBD (dsRBD along the x-axis and NTE along the y-axis) acting as a bi-directional interacting adaptor.

In order to provide a more accurate picture into how dsRBD cooperates with the other domains of hDus2 to recognize tRNA, we undertook to build a model of the full length enzyme. However, although we have the X-ray structures of the isolated domains (27), namely TBD + HD in one hand and dsRBD on the other hand, we do not know how they are positioned in the full-length enzyme. In addition, dsRBD is linked to HD by a long linker of eighteen amino acids (residue 334–351), predicted as flexible and which could promote high conformational dynamics of the protein. To test the flexibility of hDus2 in solution we performed small angle X-ray scattering experiments coupled to size exclusion chromatography (SEC-SAXS) (Figure 5A and Supplementary Figure S9D). The enzyme showed an Rg of 31 Å with a sharp distance distribution (Supplementary Figure S9E). Indeed, the calculated Kratky plot demonstrates that the protein is globular with limited flexibility rather than a multi-domain highly dynamic macromolecule (Supplementary Figure S9F). To further assess the conformational sampling of hDus2, an ensemble optimization method based on EOM was used to generate a minimal model that best fits the experimental SAXS curve deriving from a pool of 10 000 starting models. A minimal ensemble of three models was consistently identified from independent runs as best fitting the SAXS curve (Figure 5A, insert). Superposition of those models shows that the relative orientation of TBD + HD and dsRBD is conserved with limited degree of freedom most likely a consequence of intramolecular protein-protein interactions between domains. Again, this is in fair agreement with the Kratky plot suggesting limited flexibility. In these models the C-terminus forty residues populates different conformations consistent with the predicted intrinsically disordered nature of this region.

### dsRBD cooperates with TBD + HD domains for specific tRNA recognition

As hDus2 behaves as a rigid protein, with the exception of its C-terminal part, which is not important for tRNA binding (27), we superimposed our SAXS model of the full enzyme on the X-ray structure of DusA from *Thermus thermophilus* in complex with tRNA^Phe^ (31), a bacterial orthologue of hDus2 that catalyzes the same modification without dsRBD. Surprisingly, the dsRBD is perfectly positioned to interact with the acceptor stem and T/Ψ stem (Figure 5C and D), which is consistent with both NMR and SAXS data on dsRBD/tRNA complex. Interestingly, in this model, the enzyme recognizes almost the entire tRNA except for the anticodon stem-loop. To validate this binding mode, we carried RNase protection assay on a transcribed tRNA and its shorter variant lacking the anticodon stem, tRNA^ΔACS^ (Supplementary Figure S12A and B). As seen in Supplementary Figure S12C–E, hDus2 has the same affinity for both RNAs suggesting that the anticodon stem is not involved in hDus2 binding. Furthermore, tRNA bound to hDus2 seems to be less protected from RNase cleavage than tRNA^ΔACS^ as evidenced from RNase cleavage kinetics (Supplementary Figure S12F and G).

## DISCUSSION

Usually, dsRBDs serve as a RNA binding module to several enzymes that operate on RNAs lacking complex structures. Here, we unveil the molecular mechanism by which the dsRBD of hDus2 recognizes its tRNA substrate, which is characterized by a complex tertiary structure (39), to modify U20 into D20. The use of dsRBD by a tRNA-modifying enzyme remains until now a unique case. We have shown that functionalization of this dsRBD by NTE allows the emergence of a novel tRNA binding domain (dsRBD). Even though this dsRBD can bind dsRNA, it clearly exhibits a preference for tRNA (see below). From our crystallographic structures, mutagenesis, NMR and SAXS approaches together with structural information obtained on the full length hDus2 enzyme, we gathered compelling experimental evidences that enabled us to build a model for the dsRBD/tRNA complex. In the resulting model, tRNA recognition by dsRBD is mainly achieved via four regions, R1 to R4 (Figure 5D). R1 to R3 involve canonical regions that dsRBDs classically use to bind their dsRNA targets, with nonetheless some distinctive features that remain specific to hDus2, while R4 relies on NTE. R1 engages the C-terminus region of helix-α1 that should recognize the region around TΨC loop, an important tRNA region involved in tertiary interactions that are intended to maintain its L-shaped 3D-conformation. In fact, loop recognition by helix α1 C-terminus is not unprecedented and has been observed in few cases (11,13,22,23). Region R2 uses N-terminal residues from both helix α1 and α2 to jointly recognize the major groove formed between the T and acceptor arms. Again this type of binding is commonly observed in the family of RNAses III (10,23,24). Region R3 binds to the extremity of the acceptor arm via the C-terminal edge of the β1–β2 loop, which represents an unprecedented mode of interaction among dsRBD. In general, in most dsRBD, region R3 implicates the loop β1–β2, which has a generally conserved length consisting of ∼6 residues for which this size seems to be ideally suited for such a function (10). In all dsRBD/dsRNA structures available so far, the conformation of this loop points toward the RNA, and interacts with ribose moiety in the minor groove *via* both the backbone and side chain of a conserved histidine present in its conserved GPxHxx motif. However, in the case of Dus2, this loop appears shorter than the average, with only four residues, and its conformation points rather outside the dsRNA. Finally, in addition to these interactions, NTE gives birth to an R4 region of interaction that extends helix α1 interface by interacting with the T arm likely via Q367 and R361/R362. Such interactions might intend to eventually reinforce the anchoring of helix α1.

A number of recent studies have demonstrated the importance of dsRBD’s extensions in RNA binding function, while not directly contacting RNA (10,15). The case of hDus2 constitutes the first example showing that an extension is involved in direct contact with RNA. It is clear that this recognition mode provides dsRBD the capability to function as an unprecedented bi-directional molecular ruler along the x axis, with the intervention of the canonical regions R1 to R3, and along the y axis via R1 and R4 of the NTE (Figure 5D). As a result, such a type of binding increases the interaction surface, which is expected to ultimately stabilize dsRBD grips on tRNA. The location targeted by dsRBD appears to be close to the modification site and is the region that carries the longest segment of RNA duplex structure in tRNA. As it ensures the major driving force in the substrate recognition reaction (34), this domain might facilitate the positioning of the catalytic domain in a cooperative mode as in the case of other dsRBD-containing enzymes (25,40) (Figure 5D). It should be mentioned that dsRBD domains have a major contribution to their binding from electrostatic interactions and therefore it is often the case that individual mutations have minor effects on binding as we observed in the case of hDus2. Wang *et al* showed that different dsRBD proteins can have very different dynamics on structured RNA substrates and that mutations can potentially change the dynamics of an interaction (and therefore enzymatic activity) without necessarily having a large impact on binding affinity (14). As a consequence, the study of the effects of individual mutations on binding should be complemented with a study of the effects of the same mutations on uridine reduction activity to further validate our present model. Conspicuously, our work supports the idea that structural elements (here NTE and CTE) appended to the canonical dsRBD serve to upgrade the genuine fold to new functional requirement. More broadly, this could be a widespread strategy that nature has selected to recycle promiscuous RNA binding modules into specific platforms.

## Supplementary Material

Supplementary Data
