# Supplementary material for: Molecular basis for transfer RNA recognition by the double-stranded RNA-binding domain of human dihydrouridine synthase 2
Source: Nucleic Acids Res. 2019 Jan 3;47(6):3117–26. doi: 10.1093/nar/gky1302 (PMC6451096; doi:10.1093/nar/gky1302)
Supplement: Supplementary Data [file gky1302_supplemental_files.docx]

**Supplementary information for: Molecular basis for transfer RNA recognition by the double-stranded RNA-binding domain of human dihydrouridine synthase 2**

**Charles Bou-Nader^1^,** **Pierre Barraud^2,3^, Ludovic Pecqueur^1^, Javier Pérez^4^, Christophe Velours^5^, William Shepard^4^, Marc Fontecave^1^, Carine Tisné^2,3^ and Djemel Hamdane^1^**

*^1^Laboratoire de Chimie des Processus Biologiques, CNRS-UMR 8229, Collège De France, Université Pierre et Marie Curie, 11 place Marcelin Berthelot, 75231 Paris Cedex 05, France*

*^2^Institut de biologie physico-chimique (IBPC), CNRS, UMR 8261 CNRS/Université Paris Diderot, 13 rue Pierre et Marie Curie, Paris 75005, France.*

*^3^Laboratoire de cristallographie et RMN biologiques, UMR 8015, CNRS, Université Paris Descartes, Sorbonne Paris Cité, Paris, France*

*^4^Synchrotron Soleil, L'Orme des Merisiers, BP 48, 91192 Gif sur Yvette Cedex, France*

*^5^Macromolecular interaction platform of I2BC, UMR9198, Centre de Recherche de Gif-sur-Yvette, France*

*To whom correspondence should be addressed:

Djemel Hamdane, Laboratoire de Chimie des Processus Biologiques, CNRS-UMR 8229, Collège de France, 11 place Marcelin Berthelot, 75231 Paris Cedex 05, France, Tel : +33-(0)1-44271278, Email : djemel.hamdane@college-de-france.fr

**Supplementary materials and methods**

**Data collection, structure determination and refinement-** Seven datasets of 75°-sweep were recorded on different locations of the same crystal of dsRBD-RNA complex to collect 360° data with partial overlap. The final dataset on the dsRBD-RNA complex was obtained by merging the 4 best datasets with xscale. The dsRBD-RNA structure was first phased by molecular replacement using phaser ([1](#_ENREF_1)) and as template 1 molecule of dsRBD (from PDB 4wft) with 1 molecule of ideal A form 11 nucleotide RNA (5’-CGAACUUCGCG-3’) generated in Coot ([2](#_ENREF_2)). Three solutions (TFZ>13) were found showing a missing second RNA strand in the a.s.u. A second round of molecular replacement was performed using a 9-nucleotide A-form dsRNA (5’-CGAACUUCGCG-3’) generated in Coot. From the 3 solutions found (TFZ>14), only one was consistent with the expected 2 base-pair inter-dsRNA hybridization. Reciprocal space refinement was carried with autoBUSTER using target restraints from the pdb 4wft for the dsRBD. After the first refinement cycle, phenix.erraser ([3](#_ENREF_3)) was used to optimize the dsRNA hybridization pattern. Model improvement was achieved by manual building into the density with Coot. TLS were applied in the last refinement run. Given the low resolution of the data and the small number of reflections in the free set, the refinement was checked by k-fold cross validation using 12 datasets with non-overlapping free sets with 300 free reflections each. Using a starting model perturbed with phenix.dynamics ([3](#_ENREF_3)) (500 steps) and resetting the B-factors, we obtained the following statistics: Rwork=0.223 ± 0.002 and Rfree=0.257 ± 0.013.

All structures of the dsRBD mutants were phased by molecular replacement using phaser and refined with Phenix ([3](#_ENREF_3)). All diffraction data collections of the dsRBD mutants were carried out on single crystals at the micro focused PROXIMA-2 beamline at the SOLEIL synchrotron (Saint-Aubin, France) at 100 K using an ADSC Quantum 315r detector for the protein-RNA structure and an Eiger X-9M for both mutants with a wavelength of 0.9801 Å. Data were indexed, processed, merged and scaled using XDS or autoPROC ([4](#_ENREF_4)).

**SAXS data collection, analysis and model generation**- All scattering intensities were collected on the elution peaks after injection on a BioSEC-3 column (Agilent) equilibrated in 25 mM Tris-HCl pH 8, 100 mM NaCl, 1 mM MgCl_2_ and 5 % glycerol to reduce radiation damage. Data were processed using FOXTROT (<https://www.synchrotron-soleil.fr/fr/lignes-de-lumiere/swing>). The radius of gyration (Rg) was calculated by the Guinier approximation in its linearity range. Ab initio shape reconstructions were performed using the ATSAS suite ([5](#_ENREF_5)) by averaging 20 independent Dammif runs using Damaver and further refined by Dammin. The tRNA^Lys3^ X-ray structure (PDB 1fir) ([6](#_ENREF_6)) and the dsRBD X-ray structure (PDB 4wft) were used and missing parts (N and C terminal termini not observed in the crystals) were generated using eom. Comparison of the theoretical scattering curves of the protein models against the experimental curves was done using Crysol. For the complex dsRBD-tRNA^Lys3^ ab initio calculation with MONSA was used to simultaneously fit the three SAXS curves of the individual proteins and tRNA in the 2:1 complex. Ten independent runs were averaged allowing positioning of the two dsRBDs relative to the tRNA. In the case of full length hDus2, the X-ray structures (PDB 4wft and 4wfs) were used and missing parts (N and C terminal extensions not observed in the crystals as well as the catalytic loop in 4wfs) were generated using eom with 10 000 random starting models refined against the experimental SAXS curve via a genetic algorithm.

**Figure S1: Human dihydrouridine synthase 2 and its dsRDB. a**, Reaction catalyzed by human Dus2 (hDus2). This enzyme carries a flavin-mononucleotide (FMN) as a prosthetic group and uses NADPH as a hydride source for reducing uridine 20 (U20) in tRNA into dihydrouridine 20 (D20). Note that hDus2 orthologues are found in all eukaryotes including plants and fungi. **b,** Crystal structure of hDus2 dsRBD (PDB, 4WFT). The N-terminal extension (NTE) is in violet, the canonical dsRBD in orange and the structured C-terminal extension in gray.

**a**


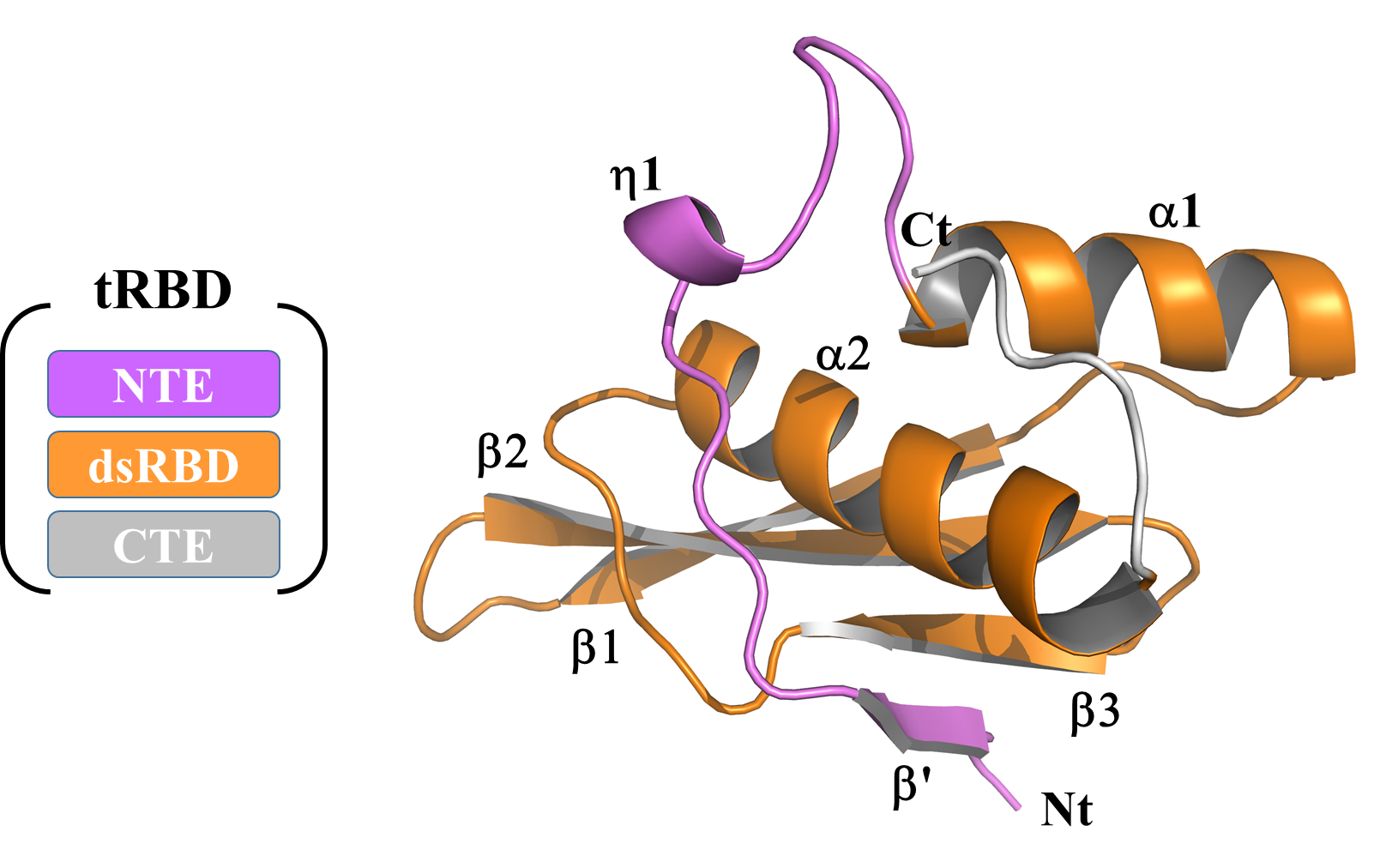


**b**

**Figure S2: Sequence alignment of dsRBDs from animal Dus2 enzymes.** In yellow are shown conserved residues across class A dsRBDs involved in RNA contacts. In red are residues implicated in folding. In blue are residues implicated in RNA recognition solely conserved in Dus2 type dsRBDs. β’ strand and η1 3_10_ helix belong to NTE of dsRBD whereas β1, β2, β3 and α1, α2 are the secondary structures of canonical dsRBD.


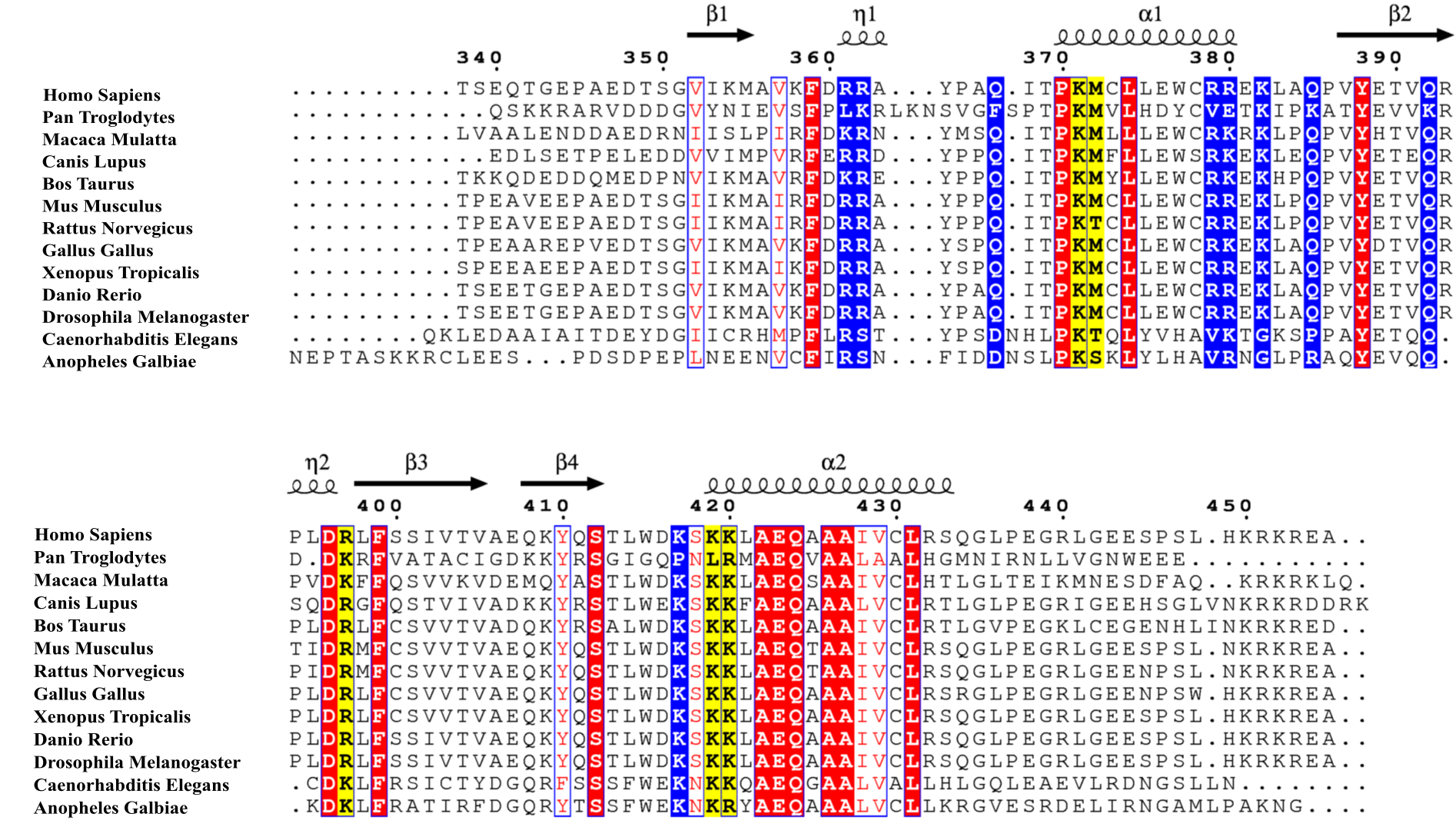


**Figure S3: X-ray structure of hDus2 dsRBD in complex with an 11-polynucleotide palindromic dsRNA. a,** Crystallographic unit cell with dsRNA in cartoon and protein in ribbon. The designed dsRNA overhang self-assembles along one axe thus favoring crystal contacts while the bound proteins further increase the order by interactions via their β-sheet. **b,** Stereoview of the 2mFo-DFc map contoured at 1σ showing the electron density in purple, the protein in grey cartoon with side chains in lines while both strands forming the dsRNA are represented in green and blue sticks respectively.


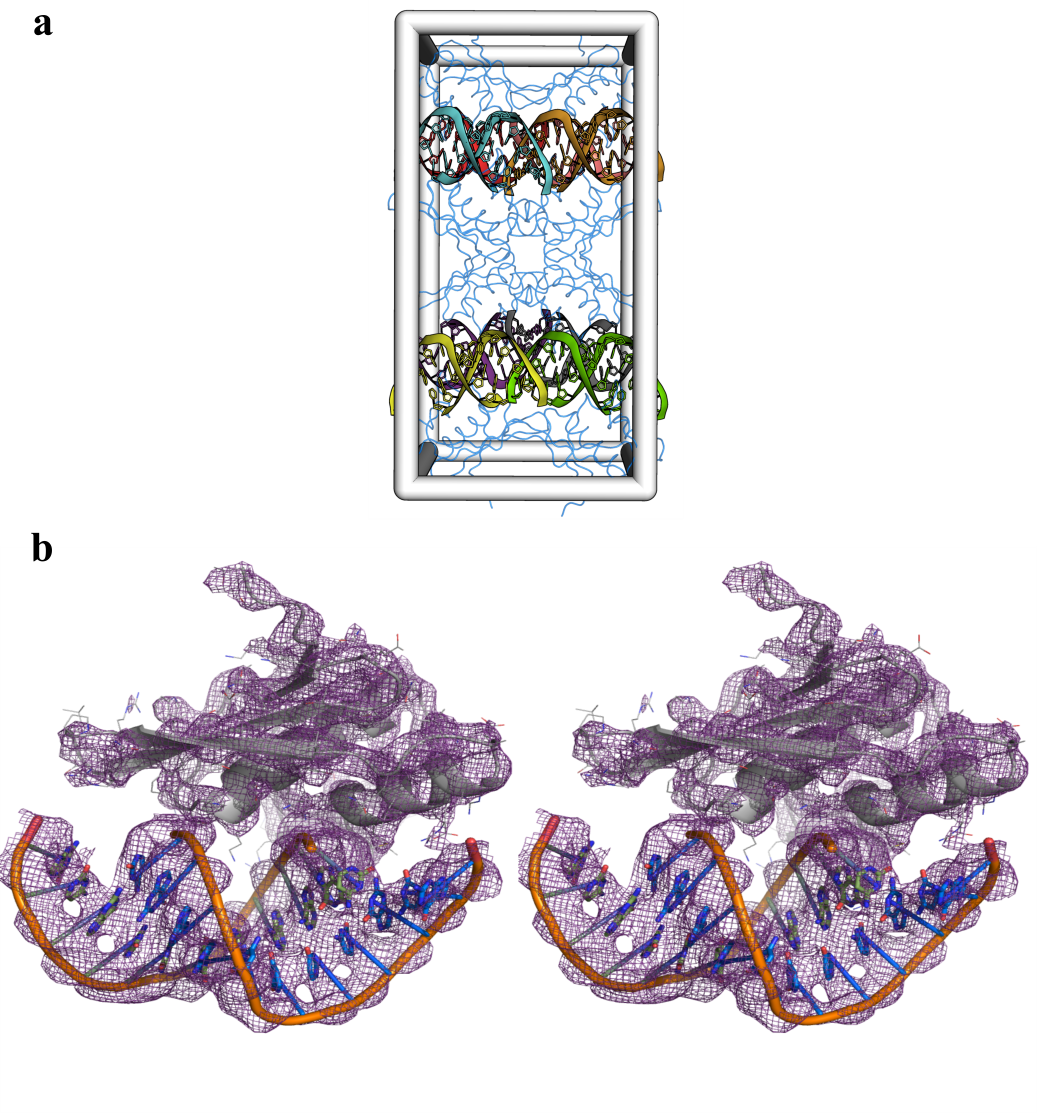


**Figure S4: Structural comparison of dsRBD free and bound to a dsRNA. a,** Structural overlay of hDus2 dsRBD bound to dsRNA (orange) and free (blue, PDB 4WFT). **b,** Zoom view showing the strands β1, β2 and the β1−β2 loop regions. **b,** Zoom view showing the residues as stick important for maintaining the hydrophobic core of the dsRBD canonical structure.


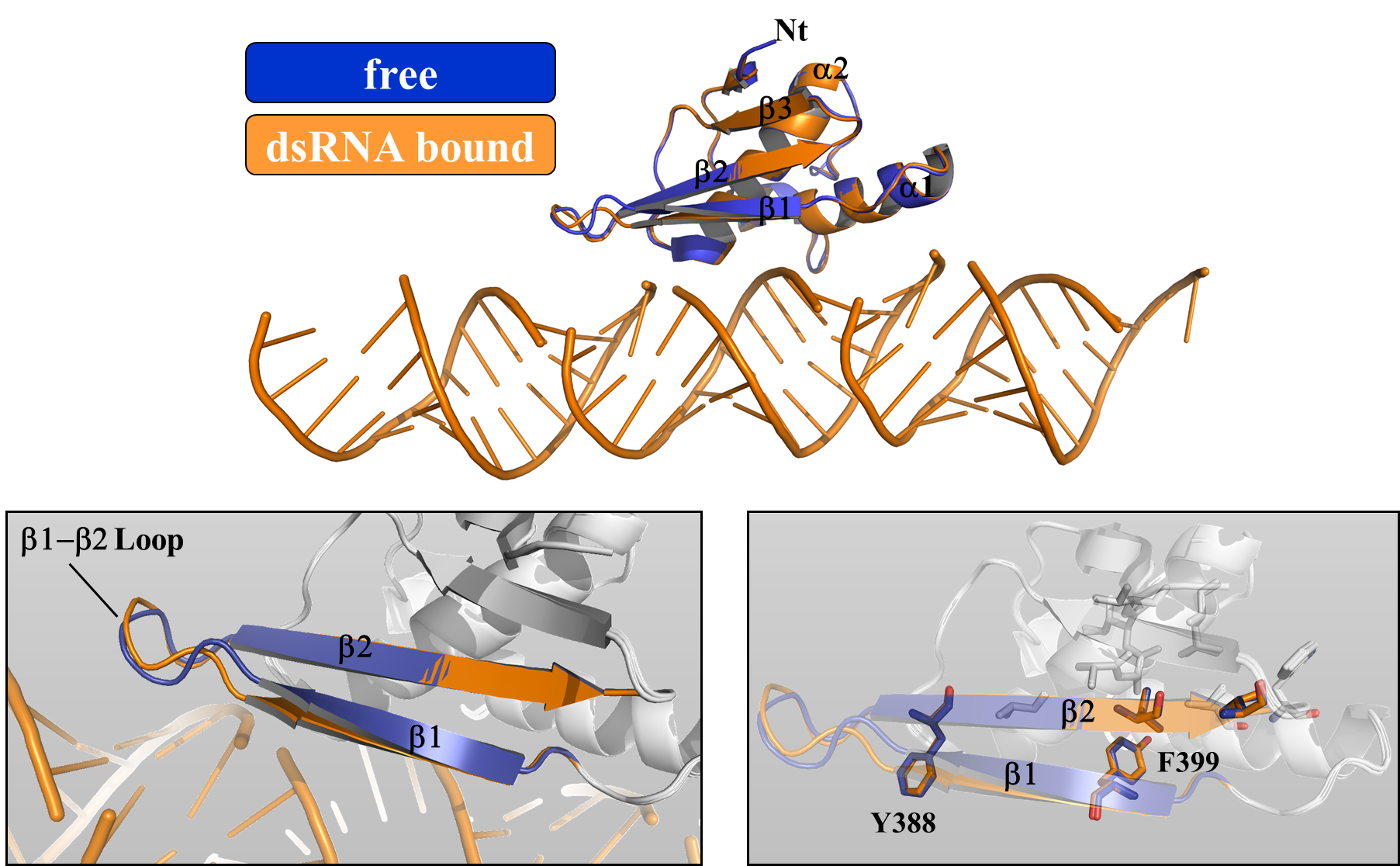


**c**

**b**

**a**

**Figure S5: Electrophoretic mobility shift assay experiments used to determine apparent tRNA affinities of wild-type dsRBD and its mutants.** Gel shift for the wild-type and mutants dsRBD. The bands corresponding to free and dsRDB bound tRNAs are black boxed. In all cases, 1 µM of tRNA^Lys3^ was used while the range of protein concentration is indicated in each case.


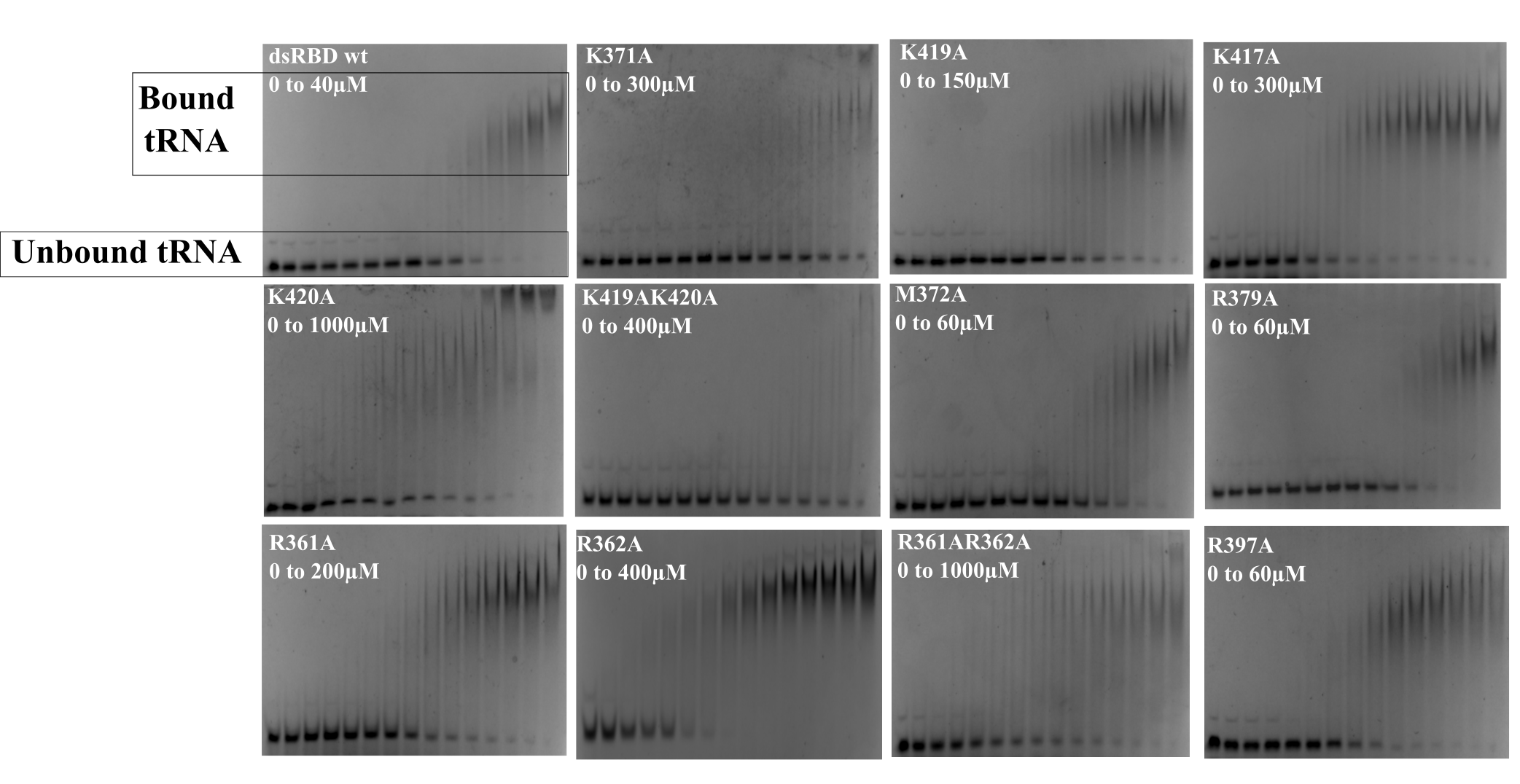


**Figure S6: dsRNA binding to wild-type dsRBD and its R361, R362, Q367A and R361A-R362A mutants. a,** EMSA for wild-type dsRBD binding to 11-polynucleotide palindromic RNA. **b,** Histogram showing the apparent Kd of wild-type dsRBD for tRNA or dsRNA. The protein has ~ 3 fold higher affinity for tRNA than dsRNA. **c,** EMSA for R361A dsRBD mutant binding to 11-polynucleotide palindromic RNA. **d,** Histogram showing the apparent Kd of R361A dsRBD for tRNA or dsRNA. The R361A mutant has a ~3.7 fold higher affinity for tRNA than dsRNA. **e,** EMSA for R362A dsRBD mutant binding to 11-polynucleotide palindromic RNA. **f,** Histogram showing the apparent Kd of R362A dsRBD for tRNA or dsRNA. The R362A mutant has a ~3 fold higher affinity for tRNA than dsRNA. **g,** EMSA for Q367A dsRBD mutant binding to 22 nucleotide dsRNA. **h,** Histogram showing the apparent Kd of Q367A dsRBD for tRNA or dsRNA. Comparison of Q367A dsRBD mutant binding to tRNA or dsRNA shows a similar affinity in both cases (~ 1.2 fold differences). **i,** EMSA for R361A-R362A dsRBD double mutant binding to 11-polynucleotide palindromic RNA. **j,** Histogram showing the apparent Kd of R361A-R362A dsRBD for tRNA or dsRNA. Comparison of R361A-R362A dsRBD mutant binding to tRNA or dsRNA shows a similar affinity in both cases (~ 1.3 fold differences).


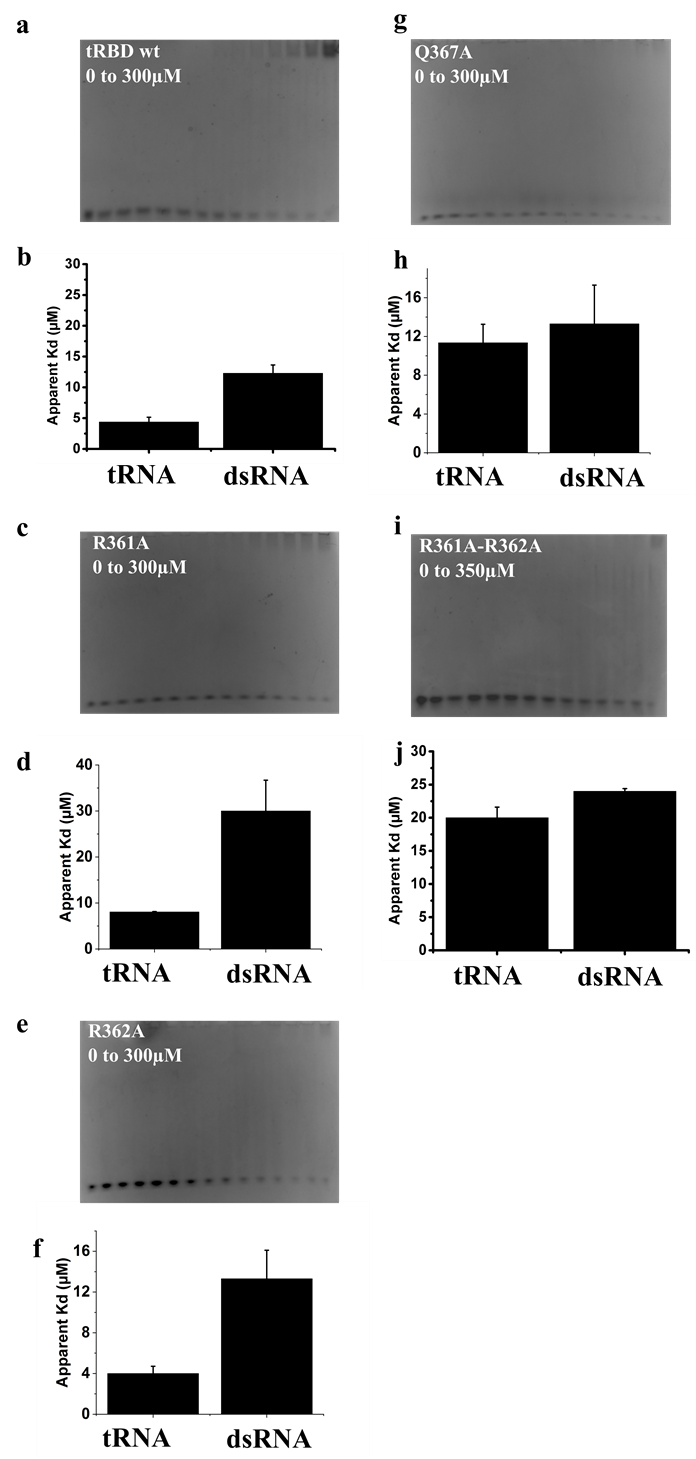


**Figure S7: NMR Backbone assignments of Dus2 dsRBD. a,** and **b,** show the (^1^H,^15^N)-HSQC assignments of ^15^N-labeled dsRBD free in solution or titrated with 1 equivalent tRNA^Lys3^, respectively. For ease of visualization only backbone assignments are shown (See also Figure 2B).

**
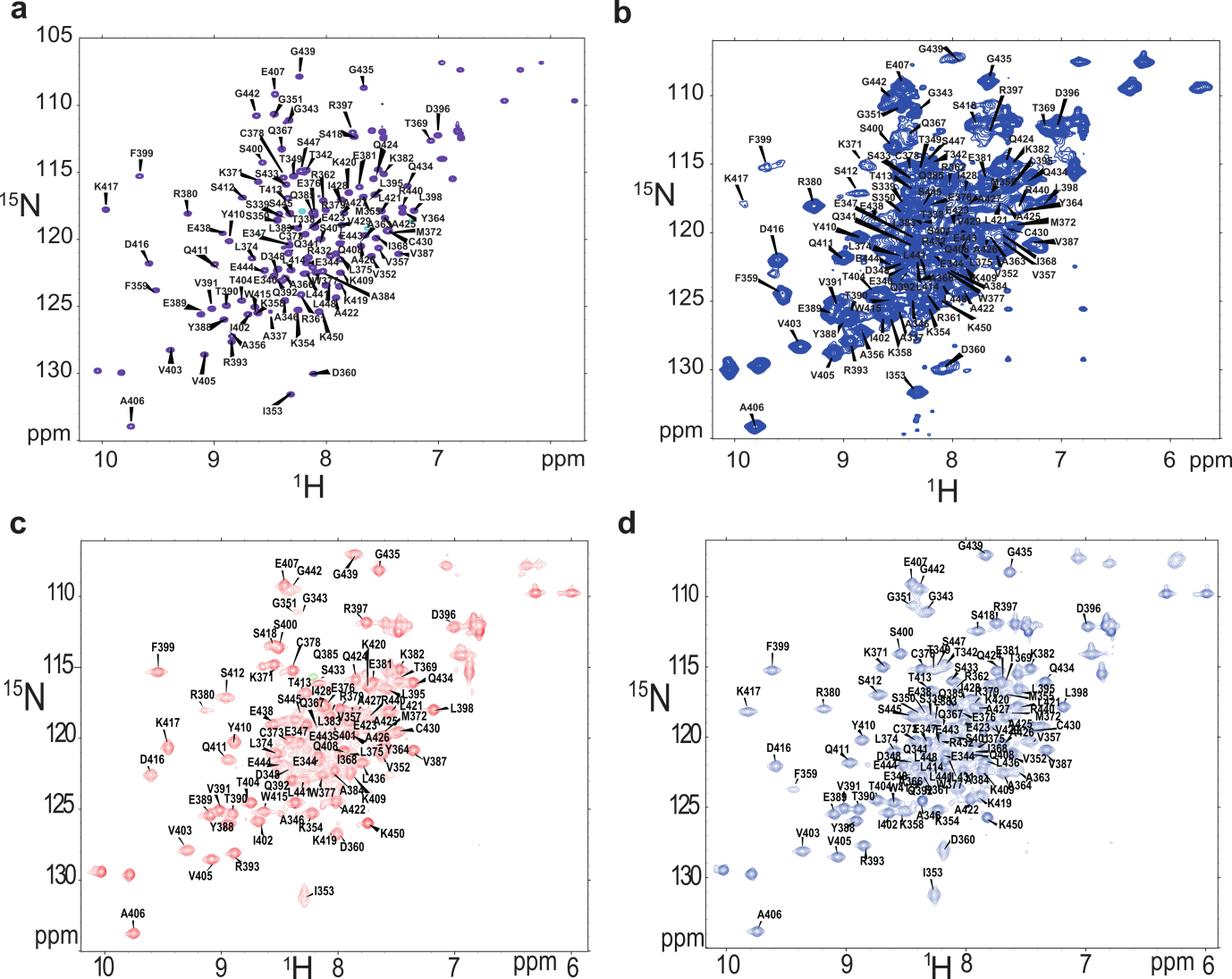
**

**Figure S8: Crystal structure of R361A-R362A and K419A-K420A dsRBD double mutants. (**a) Superposition of X-ray structures for wild-type dsRBD (pink), R361A-R362A (blue) and K419A-K420A (green) double mutants. Side chains of R361, R362, K419 and K420 in wild-type dsRBD are shown in grey sticks. (b) Structures overlay between wild-type dsRBD and R361A-R362A mutant (left); wild-type dsRBD and K419A-K420A mutant (right). The structures are colored according the RMSD value. Blue represents good structural alignment while red shows higher deviations.


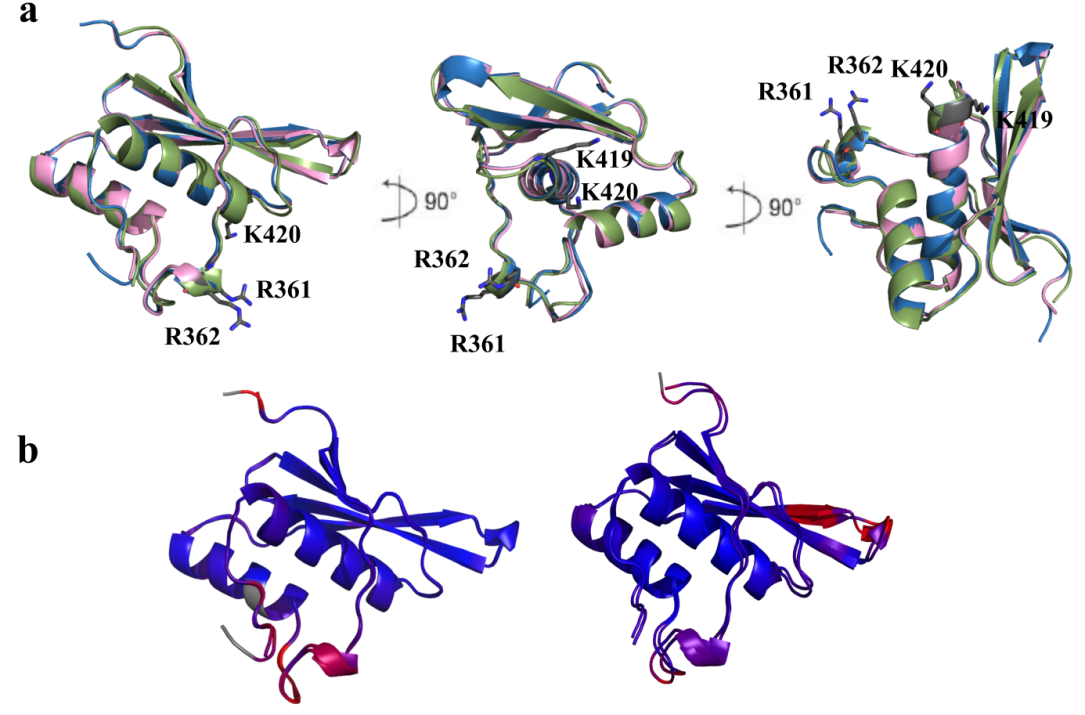


**a**

**b**

**Figure S9: Characterization of dsRDB/tRNA complex and full length hDus2 by SAXS. a-d,** SAXS data used in this study for (a) dsRBD, (b) tRNA, (c) dsRBD/tRNA complex and (d) hDus2. Insets represent the regions used for the Guinier approximation. **e,** Normalized distance distribution function calculated from the scattering profile of hDus2 using AUTOGNOM. **f,** Kratky plot of hDus2 showing that the enzyme behaves as a globular protein. **g**, model of the (dsRBD)_2_/tRNA complex. The average three-phase model (with tRNA in cyan, the first dsRBD site in green and the second in brown) is shown with three different orientations.

**b**

**a**


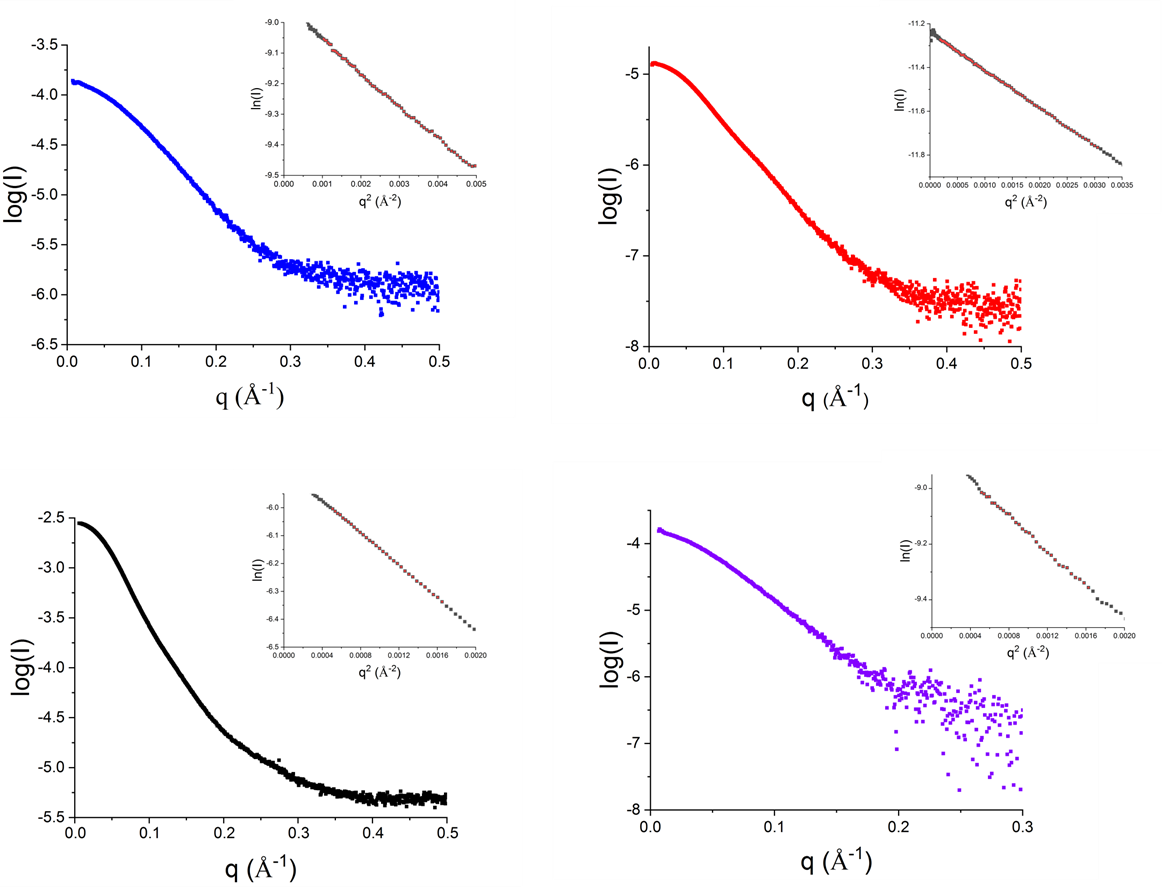


**d**

**c**


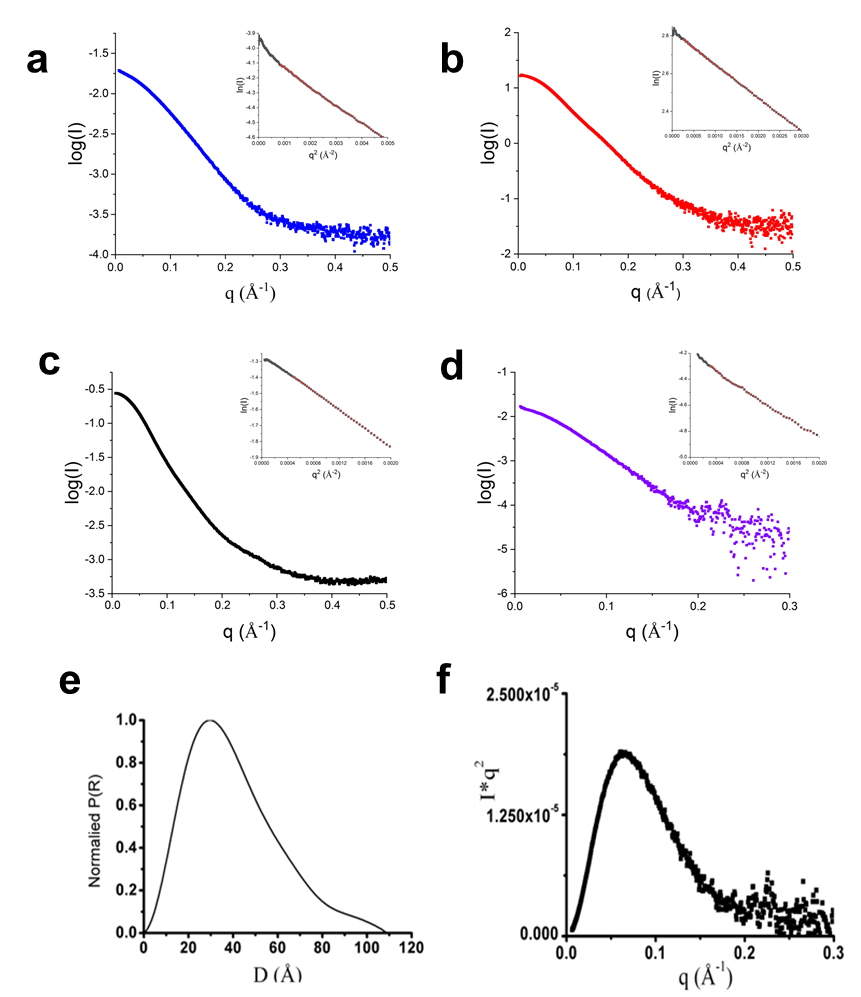


**g**


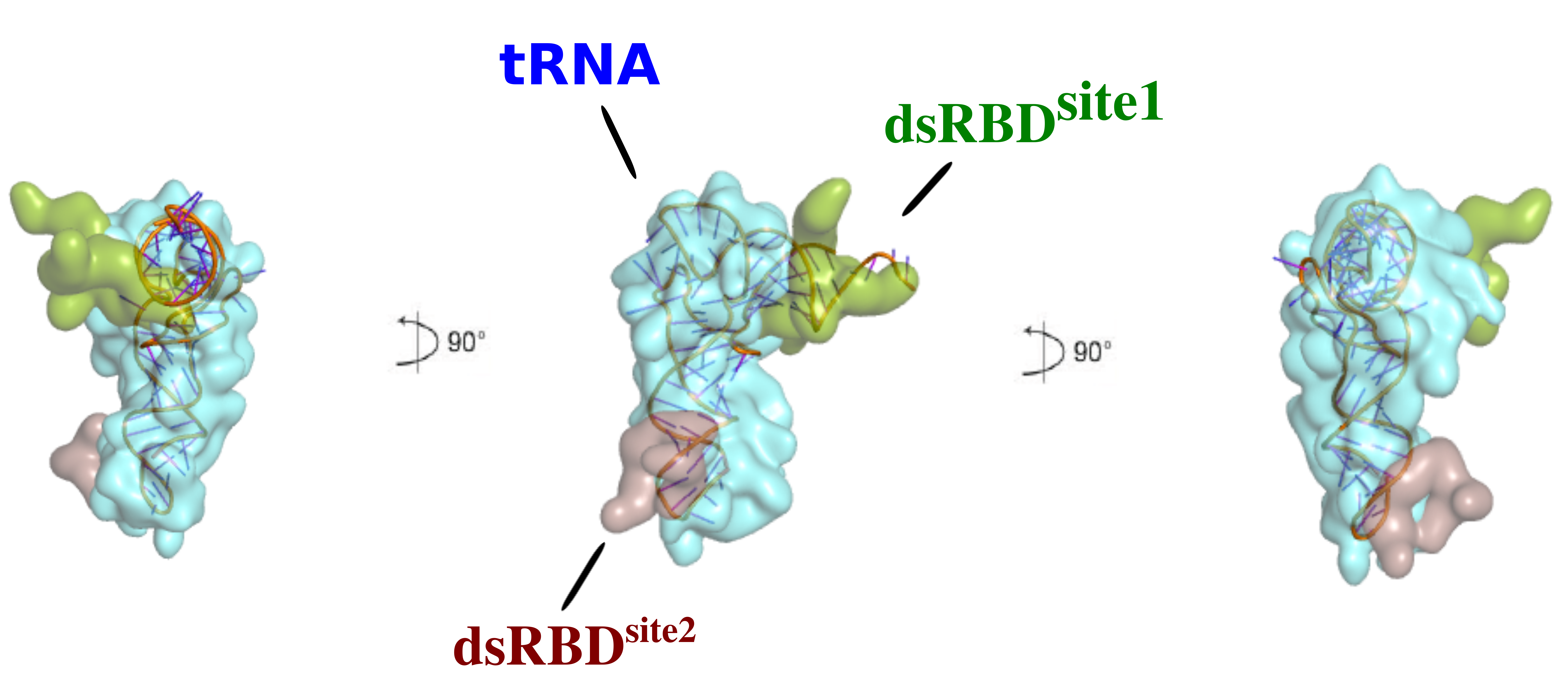


**Figure S10: SEC-MALLS analysis of dsRBD free and bound to tRNA^Lys3^. a,** SEC-MALLS of dsRBD eluting as a monomer. **b,** SEC-MALLS of tRNA^Lys3^ used in this study. **c,** SEC-MALLS of the *in vitro* reconstituted dsRBD and tRNA^Lys3^ complex showing a 2:1 protein/RNA ratio as calculated by the method protein conjugate (blue for the complex, red for the tRNA and green for dsRBD as calculated by protein conjugate).


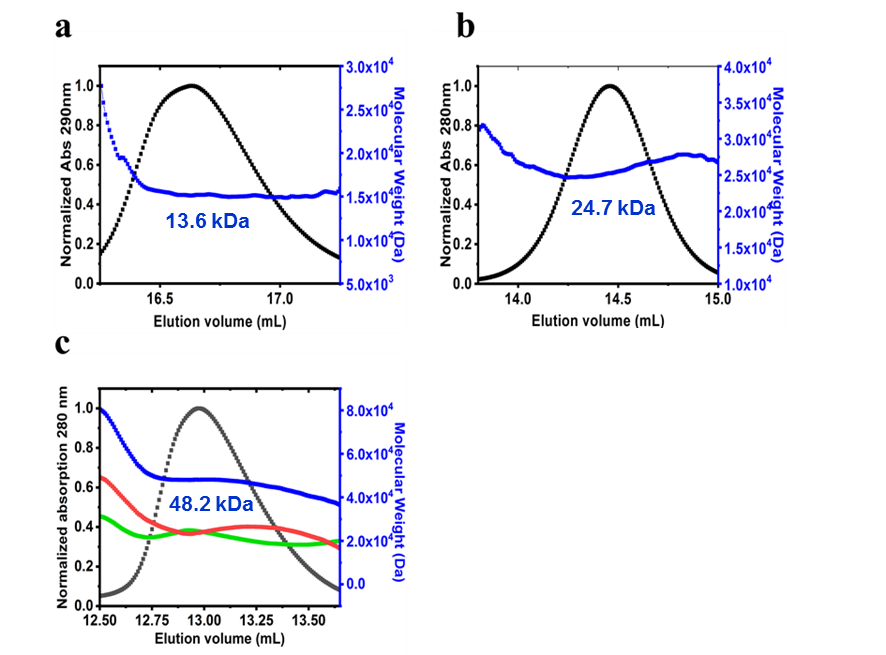


**Figure S11: SEC-MALLS analysis of full length hDus2 free and in complex with tRNA^Lys3^. a,** SEC-MALLS of hDus2 eluting as a monomer. **b,** SEC-MALLS of the *in vitro* reconstituted hDus2 and tRNA^Lys3^ complex showing a 1:1 protein/RNA ratio.


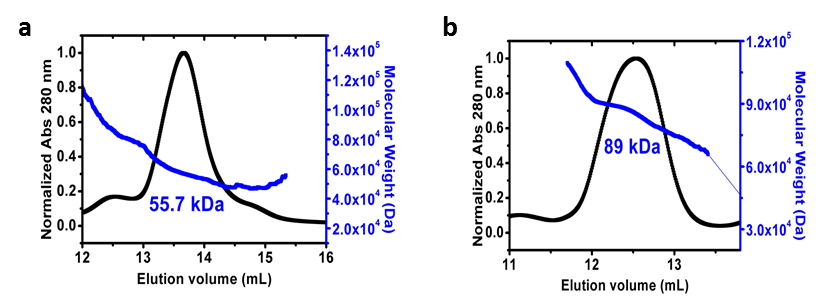


**Figure S12: RNase accessibility of bound RNA in complex with hDus2. a** and **b**, Secondary structure of tRNA^Phe^ and tRNA^Phe-ACS^ transcripts respectively. Arrows in blue represent conserved cleavage sites by RNase A between both transcripts while in red are those specific to full length tRNA. **c** and **d**, EMSA experiment with increasing concentration of hDus2 for tRNA^Phe^ and tRNA^Phe-ACS^ respectively. **e**, Calculated affinities as measured by EMSA. **f,** Degradation pattern of bound RNA in complex with hDus2. **g,** Kinetic of RNase degradation for both transcripts bound to hDus2.


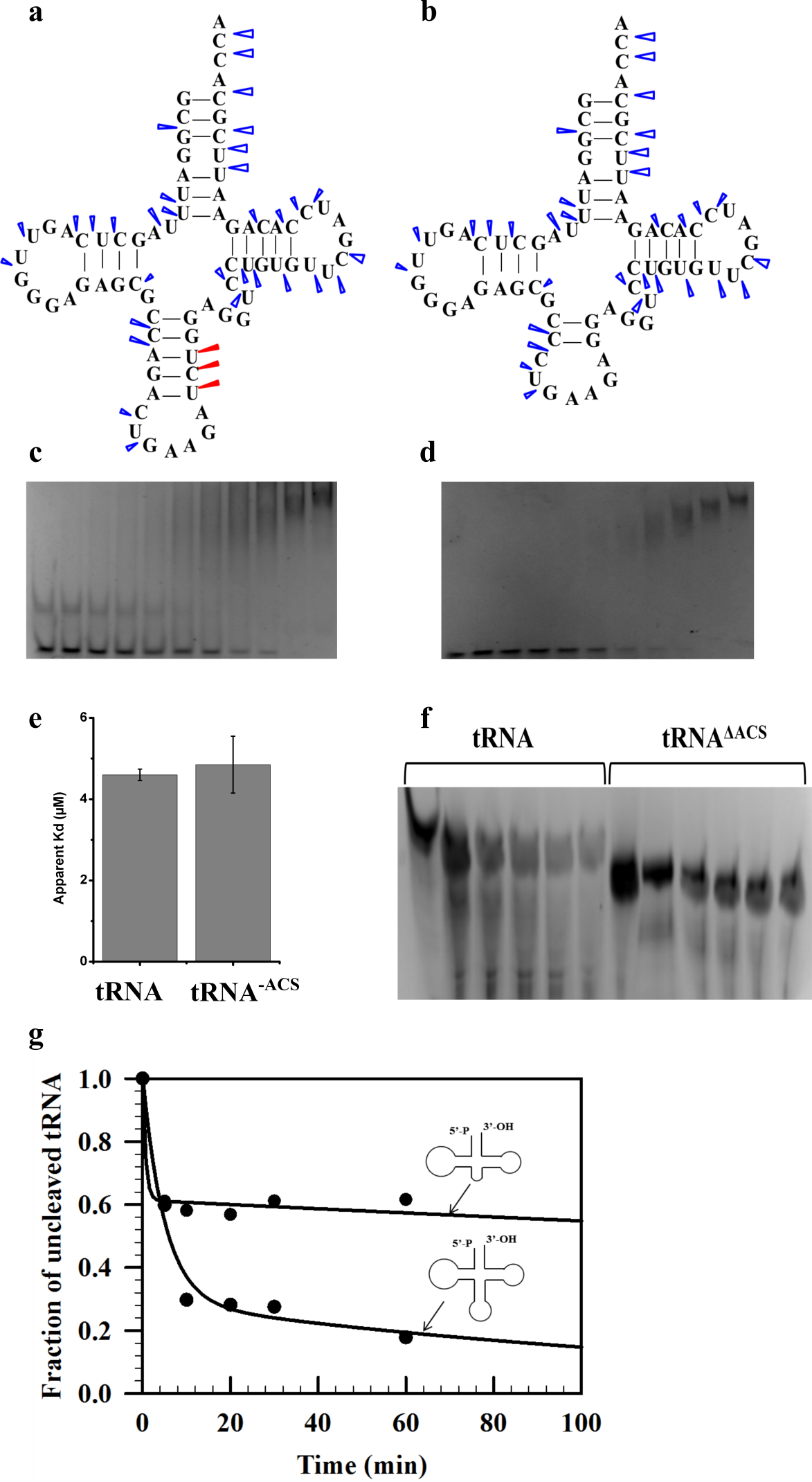


**Table S1: Summary of data collection and refinement statistics.**

|  | hDus2^dsRBD^  K419A-K420A mutant  PDB 5OC5 | | hDus2^dsRBD^  R361A-R362A mutant  PDB 5OC4 | | hDus2^dsRBD^ with 22 nucleotide self-assembling dsRNA  PDB 5OC6 | | |
| --- | --- | --- | --- | --- | --- | --- | --- |
| **Data collection** | |  | |  | |  |  |
| Space group | | P4_1_ | | R3_H_ | | C222_1_ |  |
| Cell dimensions | |  | |  | |  |  |
| *a*, *b*, *c* (Å) | | 36.17, 36.17, 75.09 | | 83.94, 83.94, 56.50 | | 58.44, 115.56, 66.62 |  |
| α, β, γ (°) | | 90, 90, 90 | | 90, 90, 120 | | 90, 90, 90 |  |
| Resolution (Å) | | 36.17- 1.89  (1.96- 1.89) | | 41.97 – 1.70  (1.76- 1.70) | | 43.65 – 3.20 (3.31- 3.20) |  |
| *R*_sym_ or *R*_merge_ | | 0.099 (1.285) | | 0.072 (1.134) | | 0.306 (1.932) |  |
| *R_meas_* | | 0.106 (1.185) | | 0.085 (1.343) | | 0.334 (2.23) |  |
| *CC_1/2_* | | 0.998 (0.578) | | 0.997 (0.394) | | 0.994 (0.31) |  |
| *I* / σ*I* | | 13.3 (1.59) | | 8.3 (0.8) | | 5.11 (0.85) |  |
| Completeness (%) | | 99.51 (96.33) | | 99.56 (100) | | 97.52 (93.44) |  |
| Redundancy | | 9.4 (7.7) | | 3.49 (3.52) | | 5.8 (3.8) |  |
|  | |  | |  | |  |  |
| **Refinement** | |  | |  | |  |  |
| Resolution (Å) | | 36.17 – 1.893 | | 41.89 – 1.71 | | 43.65 – 3.20 |  |
| No. reflections | | 7669 (709) | | 16049 (271) | | 3855 (358) |  |
| *R*_work_ / *R*_free_ | | 0.172 / 0.222 | | 0.194 / 0.215 | | 0.215/0.239 |  |
| No. atoms | |  | |  | |  |  |
| Protein | | 737 | | 746 | | 741 |  |
| RNA | | - | | - | | 468 |  |
| Ligand/ion | | 20 | | 18 | | - |  |
| Water | | 56 | | 144 | | - |  |
| *B*-factors | |  | |  | |  |  |
| Macromolecule | | 36.04 | | 34.81 | | 108.55 |  |
| Ligand/ion | | 51.54 | | 35.00 | | - |  |
| Water | | 41.22 | | 48.89 | | - |  |
| R.m.s. deviations | |  | |  | |  |  |
| Bond lengths (Å) | | 0.015 | | 0.010 | | 0.008 |  |
| Bond angles (°) | | 1.14 | | 1.06 | | 0.86 |  |

Each data set was collected from a single crystal

**Table S2: Table summarizing SAXS data used in this study**

| **Macromolecule** | **Rg (Å)** | **Dmax (Å)** |
| --- | --- | --- |
| dsRBD | 17.86 ± 0.87 | 54 |
| Human tRNA^Lys3^ | 22.76 ± 0.05 | 77.2 |
| dsRBD + tRNA^Lys3^ | 29.63 ± 0.13 | 100.3 |
| hDus2 | 30.87 ± 0.82 | 110 |

1. Adams PD*, et al.* (2010) PHENIX: a comprehensive Python-based system for macromolecular structure solution. *Acta crystallographica. Section D, Biological crystallography* 66(Pt 2):213-221.

2. Emsley P, Lohkamp B, Scott WG, & Cowtan K (2010) Features and development of Coot. *Acta crystallographica. Section D, Biological crystallography* 66(Pt 4):486-501.

3. Chou FC, Sripakdeevong P, Dibrov SM, Hermann T, & Das R (2013) Correcting pervasive errors in RNA crystallography through enumerative structure prediction. *Nature methods* 10(1):74-76.

4. Vonrhein C*, et al.* (2011) Data processing and analysis with the autoPROC toolbox. *Acta crystallographica. Section D, Biological crystallography* 67(Pt 4):293-302.

5. Franke D*, et al.* (2017) ATSAS 2.8: a comprehensive data analysis suite for small-angle scattering from macromolecular solutions. *Journal of applied crystallography* 50(Pt 4):1212-1225.

6. Benas P*, et al.* (2000) The crystal structure of HIV reverse-transcription primer tRNA(Lys,3) shows a canonical anticodon loop. *RNA* 6(10):1347-1355.
